# Dominant factors and prediction model of toppling collapse for steep cliffs in strong earthquake zones

**DOI:** 10.1038/s41598-026-55321-2

**Published:** 2026-05-31

**Authors:** Peng Zhang, Yu Xiao Yang, Naman Maimaiti, Ruiming Liu, Jili Qu

**Affiliations:** 1 College of Transportation Engineering, Nanjing Tech University, Nanjing, 210009 Jiangsu China; 2 College of Civil Engineering, Kashi University, Kashi, 844000 Xinjiang China

**Keywords:** Steep cliff hazardous rocks, Model test, 3DEC numerical simulation, Instability mode, Critical collapse vibration speed, BP neural network, Engineering, Natural hazards, Solid Earth sciences

## Abstract

Earthquake-induced collapses of steep cliffs with hazardous rock masses are a prevalent geological hazard in mountainous regions. Current methods for assessing the stability of grouped rock failures under seismic loading exhibit significant limitations. This study introduces an integrated multi-scale methodology combining scaled shaking table tests with 3DEC discrete element numerical modeling to systematically investigate the failure modes, critical collapse thresholds, and risk factors for hazardous rock formations along the China-Pakistan Highway. The approach uniquely bridges physical experimentation, numerical mechanistic analysis, and data-driven prediction to decipher complex failure mechanisms, with a focused analysis on toppling collapse. Key findings include: (1) a displacement angle threshold of ≈ 15° serves as a robust collapse indicator, outperforming conventional metrics; (2) the degree of rock weathering (fragmentation) exerts a dominant control on stability compared to joint inclination, height, and strength; (3) a developed BP neural network model effectively identifies toppling and sliding as the two predominant failure modes, utilizing joint inclination as a key discriminant; (4) collapse initiation shows a nonlinear dependence on rock geometry, where failure is accelerated by increased joint inclination or decreased rock size. Furthermore, a critical vibration velocity is established as a practical criterion for predicting toppling collapse. Factor importance analysis ranks the influencing parameters in the order: rock size > joint inclination > shape > layering configuration. The proposed thresholds, predictive criterion, and neural network model provide directly applicable tools for early warning and risk assessment, offering a refined theoretical and practical framework for mitigating seismic rockfall hazards in earthquake-prone regions.

## Introduction

**Fig. 1:**
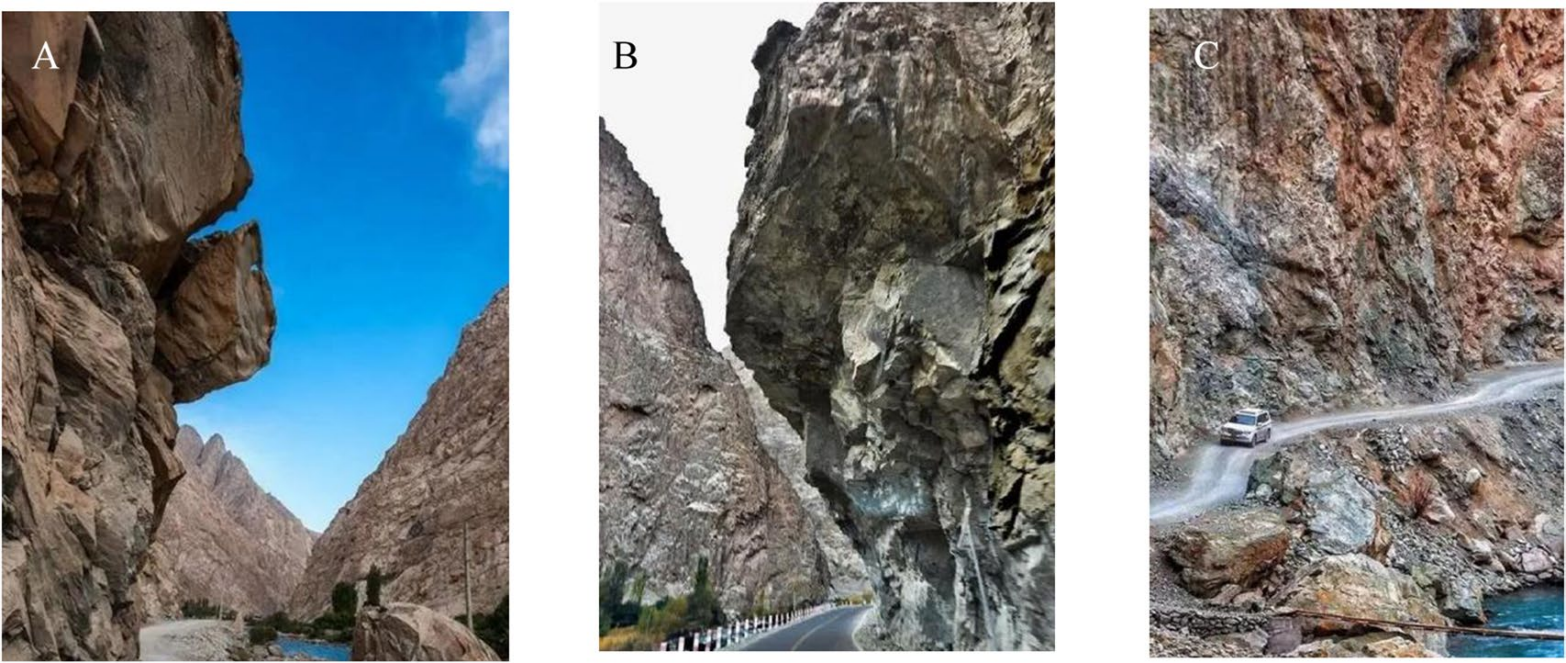
Steep cliff hazardous rock mass. (A) Typical hazardous rock mass of Gaizi River (CPEC). (B) Typical hazardous rock mass of Karakoram Highway (CPEC). (C) Typical hazardous rock mass of Tasha Ancient Road.

Steep cliff hazardous rock masses (Fig. 1) are partially detached formations with precursory failure features. They predominantly develop in structurally controlled steep slopes along the China-Pakistan Economic Corridor (CPEC)^1^. These geohazards are mainly triggered by seismic activity and intense weathering. They frequently induce cascading collapse events, which critically jeopardize transportation safety and challenge the resilience of transnational infrastructure networks^2^. The increasing frequency of these failures highlights an urgent need for a deeper mechanistic understanding and enhanced predictive modeling to protect this strategic corridor.

Contemporary studies have elucidated the complex interplay between block geometry (size, jointing characteristics), slope morphology, and dynamic loading in earthquake-induced mountain collapses. Significant progress has been made in several areas, including slope deformation metrics^2^, dynamic amplification phenomena^3^, failure mode classification^4–7^, and influencing factor analysis^1^. However, three critical knowledge gaps persist. First, there is a lack of unified stability criteria for grouped rock failures under seismic conditions. Second, the quantitative characterization of multifactor coupling effects—especially the relationship between weathering degree and joint orientation—is insufficient. Third, dynamic response models inadequately incorporate the evolution of geometric features. These limitations substantially constrain predictive accuracy for compound failure events.

Scholars Scholars have used model experiments and numerical simulations to study the stability discrimination system for group collapse of hazardous rocks under earthquake conditions. Dong et al.^5^ systematically investigated the acceleration response and damage evolution of slopes under different seismic waves through large-scale shaking table tests. Huang et al.^8^ and Li et al.^9^ focused on strong earthquakes to explore the dynamic response and damage mode of steep paragneiss slopes. He et al.^10^ studied laminated rocky slopes and clarified their seismic response characteristics and deformation mechanisms. Notably, Xin et al.^11^ first used the Hilbert–Huang transform method to reveal the spectral difference in seismic response between forwards and reverse slopes. Chen et al.^12^ applied high-speed camera technology to conduct quantitative analysis of rock mass movement behaviour.

Moreover, numerical simulation technology provides an important supplement to these study. He et al.^13^ combined RocPro3D simulations to systematically analyze the composite damage mode of hazardous rock after earthquakes. Yin et al.^14^ constructed a geomechanical model of hazardous rock clusters on the basis of discrete element theory, which revealed the destabilization mechanism under different working conditions. Yang et al.^15^ and Qi et al.^16^ simultaneously used shaking table tests and numerical simulations to comprehensively explain the damage mechanism and seismic response effects of rocky terrace slopes. Guo et al.^17^ combined model experimental results and theoretical derivations to explain the law of collapse precursor characteristics of chained hazardous rock masses. Zhou et al.^4^ and Chen et al.^18^ both investigated the damage evolution law of rocky slopes through seismic table experiments; the latter proposed the feasibility of the VMD-HT theoretical method.

The introduction of machine learning techniques has opened new research directions. Hou et al.^19^ successfully applied BP neural networks to predict tunnel lining cracks; Xiao et al.^20^ developed a PSO-BP model to significantly improve the accuracy of predicting loess infiltration depth; Liang et al.^21^ constructed a GA-BP model and demonstrated its outstanding advantages in evaluating grouting effects. Notably, Mengyu et al.^22^ confirmed through a comparative study that the BP neural network significantly outperform the traditional regression models in landslide prediction.

Nevertheless, fundamental challenges remain unresolved. First, the criticality criteria for toppling collapse lack standardization, especially under seismic conditions. Second, the relative importance of key controlling factors—such as rock fragmentation versus joint inclination—remains unquantified. Third, current models fail to adequately account for the dynamic effects of evolving geometric features during the failure process. These limitations significantly hinder the development of reliable early warning systems for infrastructure protection.

This study introduces an innovative multiscale, three-part research methodology. It integrates physical testing, numerical simulations, and machine learning approaches. Visual monitoring of the damage process is accomplished through shaking table tests and digital image correlation (DIC) technology. A discrete fracture network model based on 3DEC software is developed to accurately simulate the dynamic response of jointed rock. Additionally, a risk prediction model incorporating multiparameter coupling is established using a combination of BP neural network and deep learning. The research aims to: (i) establish a dynamic similarity-based classification system for composite failure modes; (ii) develop an energy-based stability index quantifying critical acceleration thresholds; (iii) construct machine learning-driven risk factor identification models. These advancements are expected to provide transformative solutions for geological risk mitigation along the CPEC and similar infrastructure networks globally.

## Small shaker model tests

### Test equipment and materials

**Fig. 2:**
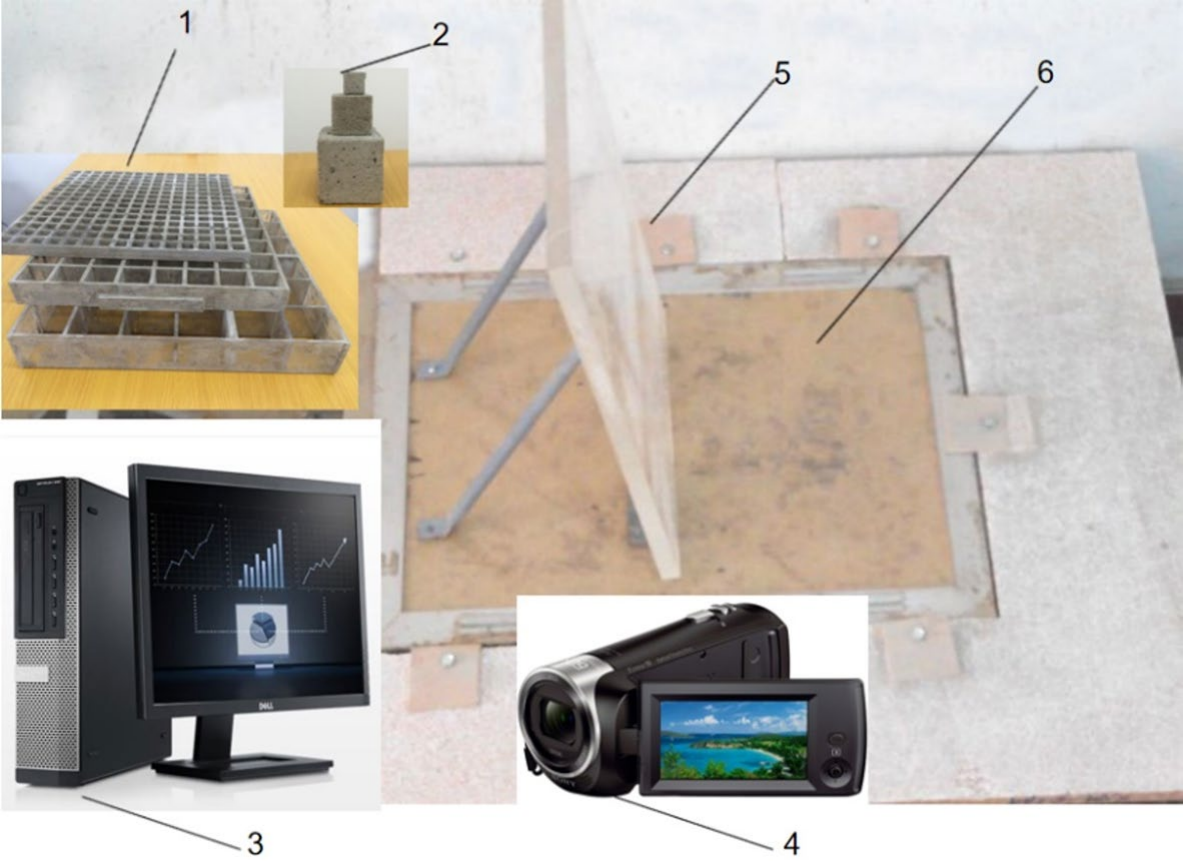
Shaking test platforms (1 - mould; 2 - rock block; 3 - seismic wave input computer; 4 - high-definition camera; 5 - nodal angle control plate; 6 - shaker).

Plexiglas was used to fabricate three sizes of cubic moulds (side lengths of 1.5 cm, 3 cm and 5 cm), casting more than 4000, 800 and 250 rock models, respectively, to simulate hazardous rock bodies with different degrees of fragmentation. The rock blocks were fabricated from a common cement-mortar mixture, primarily composed of Ordinary Portland Cement, fine sand, and water. The mixture proportion by weight was cement: sand: water = 1:2:0.45. This mix was designed to simulate the brittle failure behavior of weak to medium-strength rock. The key physical and mechanical parameters of the analog material, determined through standard laboratory tests (uniaxial compression, direct shear) on representative samples, are summarized in Table 1. The vibrating table surface was made of a 398 mm × 598 mm × 10 mm plexiglass base plate and was installed in the position of the original shear model box. To construct a support structure for the hazardous rock, a 300 mm × 600 mm × 20 mm plexiglass baffle was used; the two plates were connected through a loose leaf and reinforced with two hollow steel tubes at the back to prevent loosening due to vibration. During the test, the seismic wave parameters were input through the computer, and a video camera was used to record the whole collapse process (Fig. 2).

The model test was designed based on the Froude similarity law, which governs physical processes where gravitational forces are dominant.The scaling relationships for key physical quantities, derived from the Froude similarity law and the requirement for constitutive relation similarity, are summarized in Table 2.

**Table 1:** Composition, mixture proportion, and mechanical parameters of the analog rock material.

Property	Value	Unit	Remarks
Primary composition	Ordinary Cement, Fine Sand, Water	-	Typical cement-mortar mixture
Mixture proportion (by weight)	1 : 2 : 0.45	-	Cement : Sand : Water
Density (ρ)	2250 ± 50	kg/m^3^	Measured
Uniaxial compressive strength (UCS, σ_c_)	18 ± 2	MPa	Tested on 50 mm cubes
Young’s modulus (E)	18 ± 2	GPa	Secant modulus at 50% of peak UCS
Cohesion (c)	3.5 ± 0.5	MPa	Derived from direct shear test on mortar specimens
Internal friction angle (φ)	35 ± 2	°	Derived from direct shear test on mortar specimens

**Table 2:** Similarity ratio of the model test.

Category	Physical quantity	Symbol	Scale factor	Value
Geometry	Length	$\:{{ \alpha}}_{\mathrm{L}}$	$\:{\mathrm{L}}_{\mathrm{m}}\mathrm{/}{\mathrm{L}}_{\mathrm{p}}$	1/100
	Angle	$\:{{ \alpha}}_{ \beta}$	1	1
Material	Density	$\:{{ \alpha}}_{ \rho }$	$\:{ \rho }_{\mathrm{m}}\mathrm{/}{ \rho }_{\mathrm{p}}$	1
	Unit weight	$\:{{ \alpha}}_{ \gamma }$	$\:{ \gamma }_{\mathrm{m}}\mathrm{/}{ \gamma }_{\mathrm{p}}$	1
	Elastic modulus	$\:{{ \alpha}}_{\mathrm{E}}$	$\:{\mathrm{E}}_{\mathrm{m}}\mathrm{/}{\mathrm{E}}_{\mathrm{p}}$	1
	Poisson’s Ratio	$\:{{ \alpha}}_{ \mu }$	1	1
Dynamic loading	Acceleration	$\:{{ \alpha}}_{\mathrm{a}}$	1	1
	Time	$\:{{ \alpha}}_{\mathrm{t}}$	$\:{{ \alpha}}_{\mathrm{L}}^{\mathrm{1/2}}$	1/10
	Frequency	$\:{{ \alpha}}_{\mathrm{f}}$	$\:{{ \alpha}}_{\mathrm{L}}^{\mathrm{-1/2}}$	10
	Velocity	$\:{{ \alpha}}_{\mathrm{v}}$	$\:{{ \alpha}}_{\mathrm{L}}^{\mathrm{1/2}}$	1/10
	Displacement	$\:{{ \alpha}}_{ \delta }$	$\:{{ \alpha}}_{\mathrm{L}}$	1/100

Note: The assumption of $\:{{ \alpha}}_{ \rho }\mathrm{=}{{ \alpha}}_{\mathrm{E}}\mathrm{=1}$ is an idealization for material similarity. The cement-mortar used in this study was designed to approximate these requirements for weak rock behavior.

### Test methods

On the basis of the tectonic characteristics of blocky hazardous rocks in steep cliffs^17^, this study adopts a simplified test framework that focuses on four major influencing factors: hazardous rock fragmentation, joint inclination, hazardous rock height and seismic intensity. Cube models of different sizes were used to simulate the degree of hazardous rock fragmentation: 1.5 cm (severely fragmented), 3 cm (moderately fragmented) and 5 cm (mildly fragmented). The knuckle inclination (60°, 70°, 80°, and 90°) was adjusted by means of a Plexiglas plate connected by a loose leaf, and different stacking heights (27 cm, 30 cm, 33 cm, and 36 cm) were used to simulate the size of the hazardous rock. Seismic loads were applied by a shaking table, and different magnitudes (0.05 g, 0.1 g, and 0.2 g, corresponding to magnitude 6, 7 and 8 earthquakes, respectively) were simulated by sine waves(Table 3). The tests were completed by relying on the shaking table system at the Institute of Geotechnical Engineering, Nanjing Tech University, to ensure that the test conditions were consistent with the actual engineering background^23–26^.

**Table 3:** Frequency and amplitude values of sine waves.

Earthquake intensity	Accelerations a	Frequency f/Hz	Amplification A/mm
8	0.2 g	3	5.5
		3.5	4
7	0.1 g	3	2.76
		2	6.2
6	0.05 g	2	3.1
		1.5	5.5

Note: acceleration a = 4Af^2^π^2^.

To quantitatively capture the displacement field and deformation evolution during shaking, Digital Image Correlation (DIC) was employed. Two high-resolution cameras (Model X, Resolution Y) were positioned orthogonally to the model face, recording at 60 frames per second. The rock block surfaces were speckled with a high-contrast random pattern to facilitate tracking. Displacement accuracy of the DIC system was calibrated to be within ± 0.05 mm, and strain measurement precision was better than 0.1%. The acquired images were processed using GOM Correlate software (v2020), which provided full-field displacement vectors and time-history data for key points, enabling the calculation of the displacement angles discussed in section “Test analyses”.

### Test scheme

During the pretest stage, the most representative test conditions were determined by testing several sets of parameter combinations (including different block stacking methods, heights, inclination angles and vibration parameters). The model demonstrated good stability and was representative when 3 cm blocks (10 layers stacked with a total height of 30 cm), a joint inclination angle of 80°, and a vibration intensity of 0.1 g were used. On this basis, the baseline test programme was established, and 14 groups of comparative tests (3 groups of block sizes, 4 groups of heights, 4 groups of nodal angles, and 3 groups of vibration intensities) were designed using the one-way variable method, as shown in Table 4.

**Table 4:** Scheme of model test.

No	Block size/cm	Hazardous rock height/cm	Sectional inclination/(°)	Vibration intensity/(g)
1	1.5	30	80	0.1
2	3	30	80	0.1
3	5	30	80	0.1
4	3	27	80	0.1
5	3	33	80	0.1
6	3	30	80	0.1
7	3	36	80	0.1
8	3	30	60	0.1
9	3	30	70	0.1
10	3	30	80	0.1
11	3	30	90	0.1
12	3	30	80	0.05
13	3	30	80	0.1
14	3	30	80	0.2

### Test analyses

**Fig. 3:**
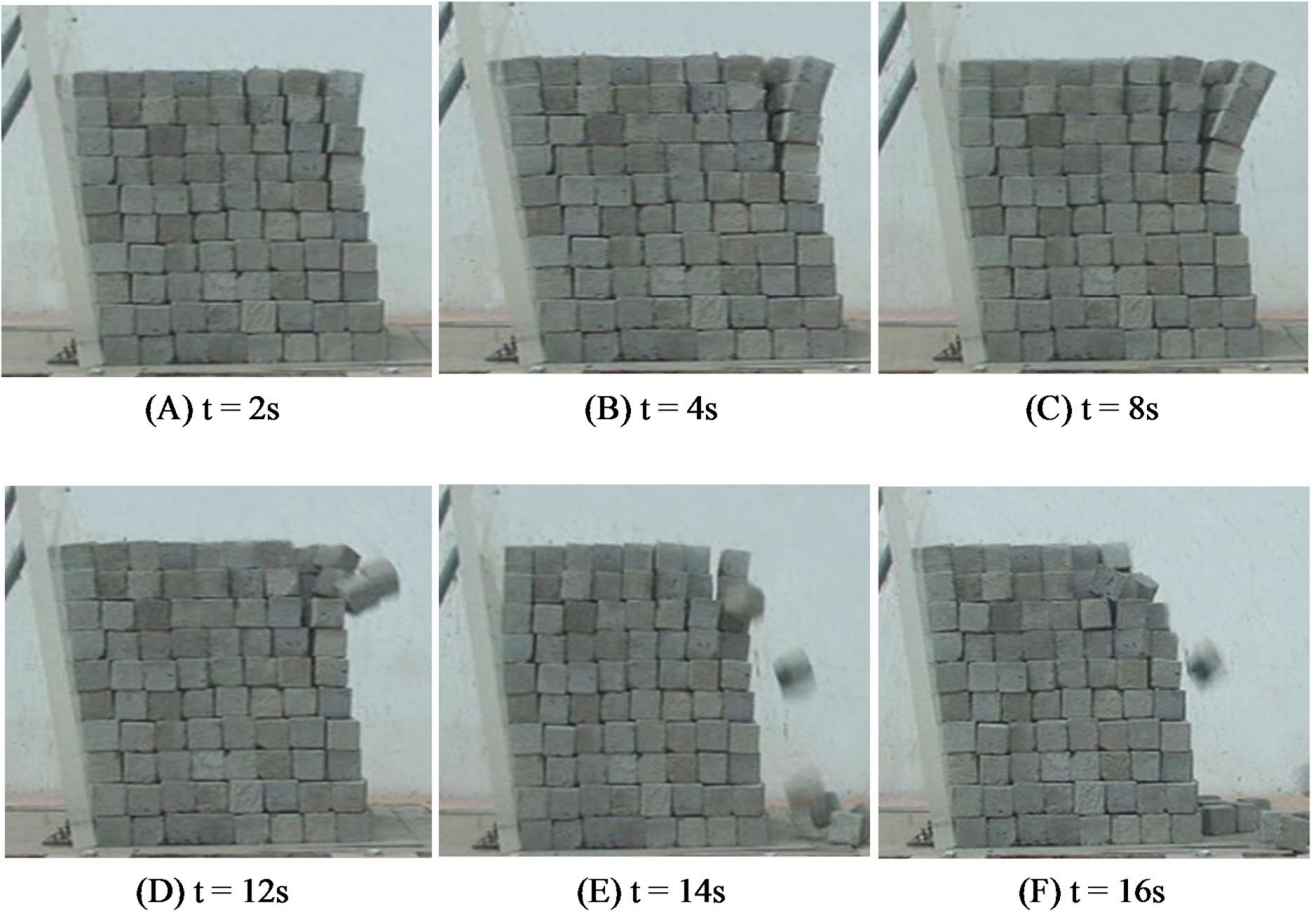
Failure forms at different times of the block collapse.

**Fig. 4:**
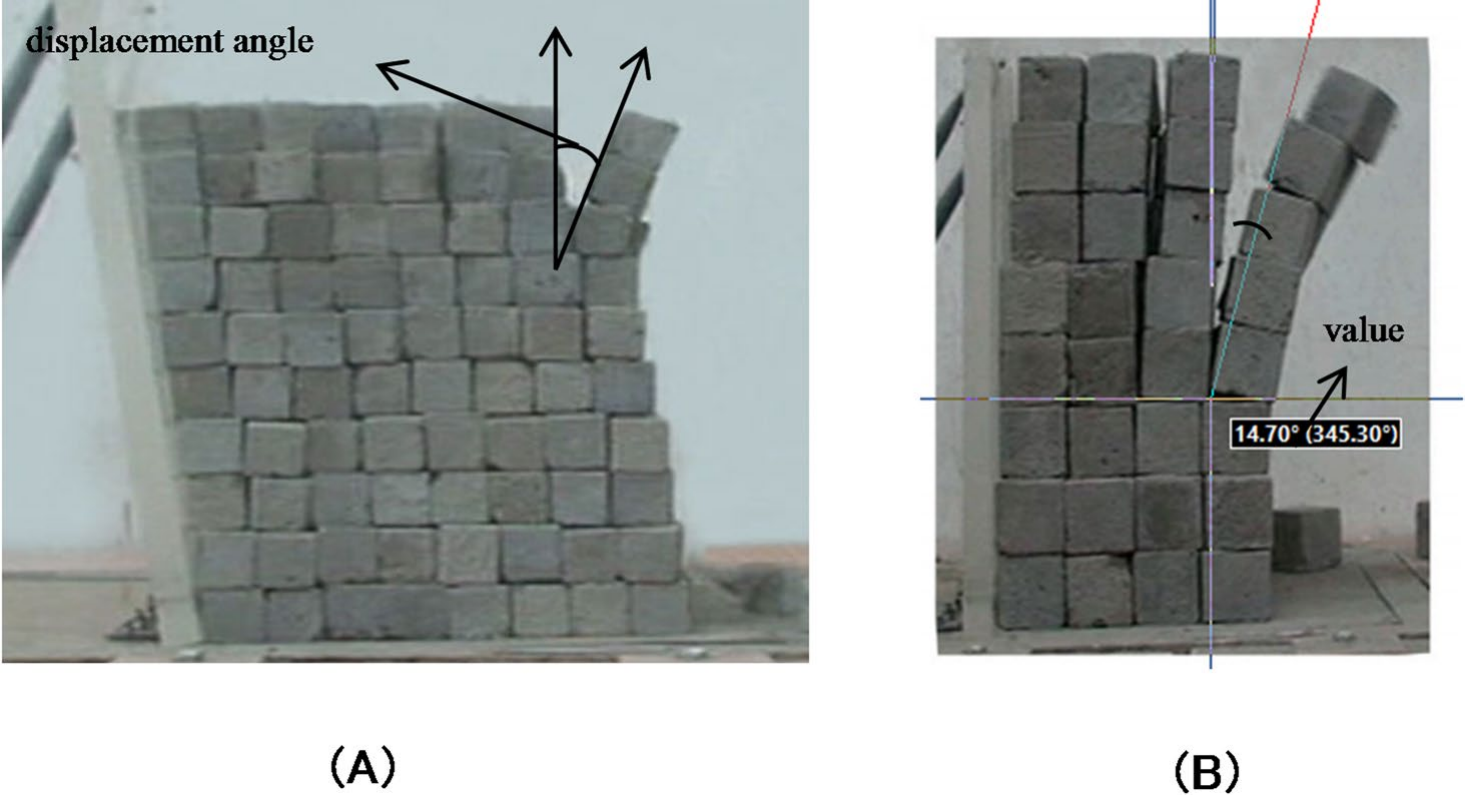
Definition and measurement of the displacement angle. (A) Schematic diagram defining the displacement angle as the angle between the major axis of the rocking rock column and the original joint line. (**B**) A practical measurement screen of Pickpick software, obtaining the displacement angle value(14.7°) directly.

From the shaker model test, observations indicate that collapse usually starts at the top of the block at the outermost edge of the hazardous rock body (Fig. 3) and then gradually expands. To quantitatively characterize this failure initiation, we introduce the displacement angle. As schematically defined in Fig. 4A, it is the angle between the major axis of the outermost rocking rock column and the original (pre-vibration) joint line. This angle was directly measured from high-frequency video recordings of the tests using the image analysis software PickPick, which provides precise angular measurements on screenshots. The use of the displacement angle as a criterion for collapse has significant advantages: (1) it helps eliminate the effects of height differences and achieve a uniform evaluation of blocks at different locations; (2) compared with displacement monitoring, the range of changes in the angular parameter is more concentrated, which enables a more accurate identification of the critical point of collapse. Therefore, in this study, the displacement angle is used as a key index to assess the stability of a block.

**Fig. 5:**
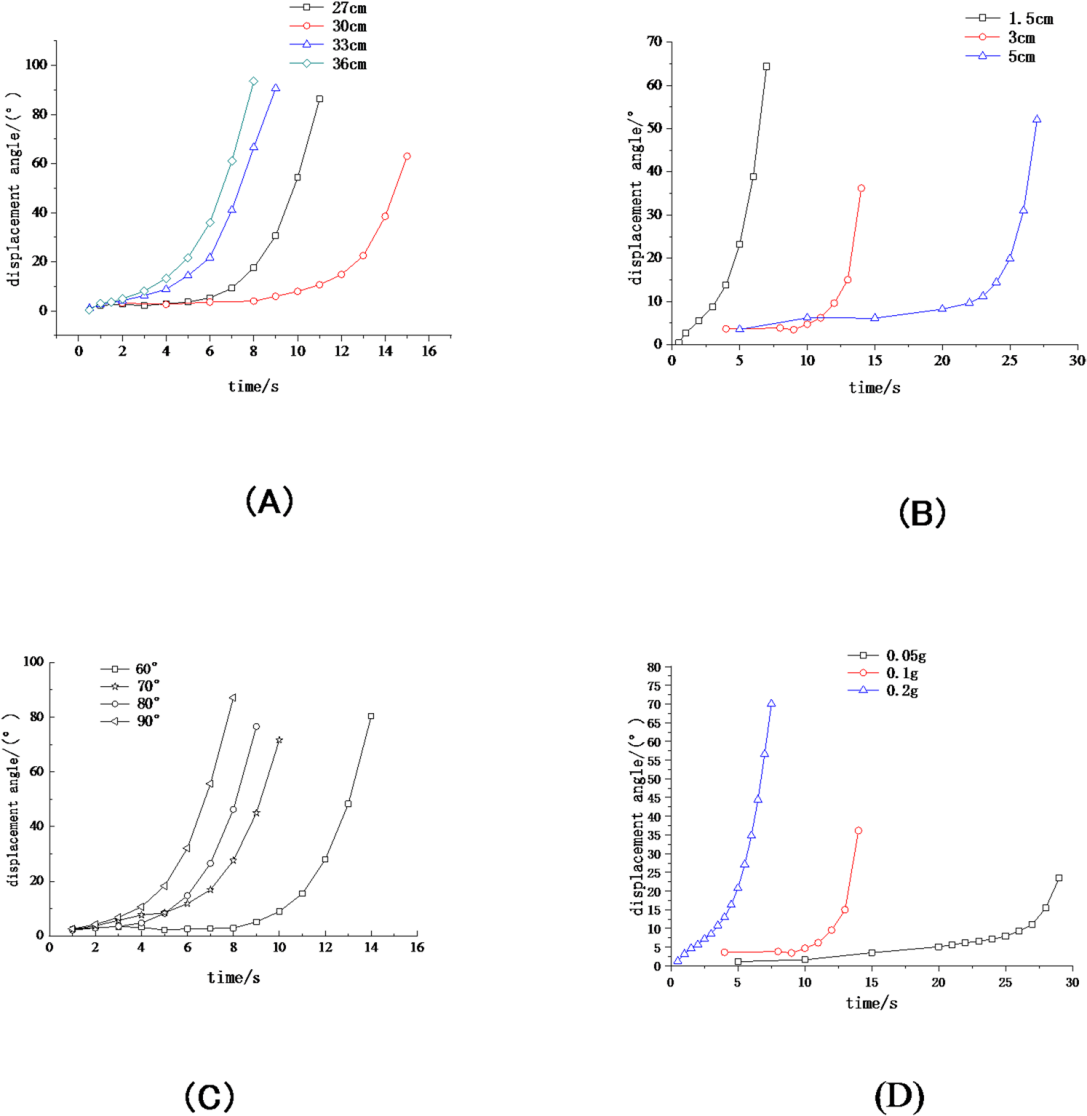
Displacement angle‒time curves showing avalanche displacement of hazardous rockes under different conditions. (A) Different hazardous rock heights; (B) varying fragmentation degrees; (C) different joints; (D) varying seismic intensities.

After opening the screenshot, launch the Pickpick software. Select the protractor function, and the software will automatically hide. Click the angle vertices on the screenshot, then drag out the two angle sides. The software will directly display the angle values. Figure 4B illustrates the practical measurement screen of software, obtaining the displacement angle value directly of any moment before the collapse. The time-history curve of the displacement angle (Fig. 5) was constructed from such measurements. The inflection point of this curve, where the angle begins to rapidly increase, was identified as the collapse initiation time, and the corresponding angle value was recorded as the collapse displacement angle.

The test results reveal that there was a significant correlation between the collapse angle of hazardous rocks and the influencing factors (Fig. 5). Specifically, the collapse angles corresponding to the degrees of fragmentation (1.5 cm, 3 cm and 5 cm) were 9°, 11° and 15°, respectively, which were obviously positively correlated; the collapse angles corresponding to the hazardous rock heights (27–36 cm) and the joint angles (60–90°) fluctuated within the ranges of 10–18° and 14–16°, respectively, without obvious monotonic changes. The seismic intensity (0.05–0.2 g), on the other hand, caused the collapse angle to vary between 9–15°. Notably, the collapse angles under all working conditions were generally concentrated at approximately 15°, and it can be assumed that the collapse angle threshold of hazardous rock was approximately 15°, with the strongest connection between rock fragmentation.

A comprehensive analysis revealed that the degree of hazardous rock fragmentation (degree of weathering) is the most critical factor affecting collapse, which has a stable and positive correlation with the collapse angle, whereas the influences of other factors are relatively complex and nonlinear. This result suggests that long-term effective weathering prevention measures should be prioritised in practical engineering protection to significantly reduce the risk of hazardous rock failure. Moreover, the collapse angle threshold of 15° can provide an important reference basis for engineering monitoring and early warning.

## Study area and characteristics of hazardous rock masses

### Geological background of the study area

The study focuses on steep cliff sections along the China-Pakistan Highway (CPEC domestic segment), traversing the rugged terrain of the western Kunlun Mountains in Xinjiang, China. This region is characterized by intense neotectonic activity, high seismic intensity (historically subjected to earthquakes with magnitudes up to M ~ s~ 8.0), and severe environmental weathering, which collectively predispose slopes to widespread hazardous rock development. The lithology of the target cliffs predominantly consists of Paleozoic to Mesozoic metamorphic rocks, including gneiss, schist, and slate, interbedded with hard, brittle granitic intrusions. These rocks have been subjected to extensive tectonic fracturing, resulting in well-developed, persistent joint sets that define the structural framework for potential rockfalls.

### Field characterization of hazardous rock masses

To ensure the experimental and numerical models accurately reflect field conditions, comprehensive field surveys were conducted along several representative high-risk cliff sections. The characterization involved detailed structural mapping, drone-based photogrammetry, and statistical analysis of discontinuity networks using scanline surveys.

**Fig. 6:**
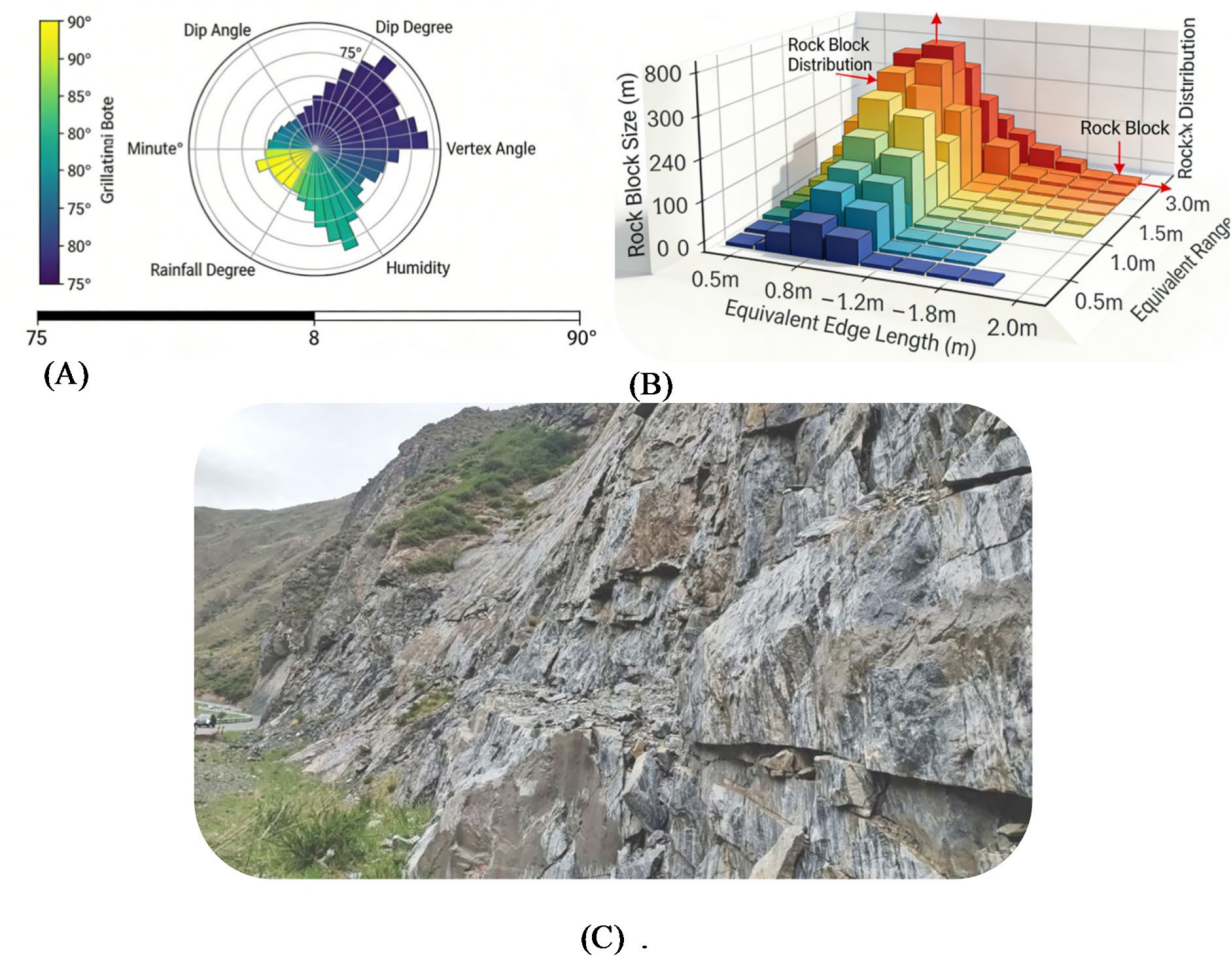
Characterization data of Hazardous Rock Masses. (A) Rose diagram of measured joint dip angles. (B) Histogram of measured hazardous rock block sizes. (C) Field photographs showing typical steep cliffs and hazardous rock morphologies.

1. Joint Fabric and Inclination: The stability of the cliffs is primarily controlled by one dominant, steeply dipping joint set. Statistical analysis of 152 measured discontinuities across three scanlines revealed that the dip angle of this controlling joint set is predominantly concentrated between 75° and 90° (Fig. 6-A), with a mean value of approximately 83°. This near-vertical structural fabric is the primary geological precondition for toppling failure mechanisms.
2. Characteristic Size of Hazardous Rock Blocks: The spacing of the dominant joint set, cross-cut by secondary fractures, defines the in-situ block size. Measurements of 87 isolated or semi-detached hazardous rock blocks indicated a wide but characteristic size distribution. The equivalent block side length (calculated from measured dimensions) predominantly ranges from 0.4 m to 1.8 m (Fig. X-b). Blocks within this size range were frequently observed in a critical, unstable state, posing direct threats to the highway.
3. Weathering and Fragmentation Degree: The rock mass in the study area exhibits moderate to high degrees of surface and subsurface weathering. Field assessments using standardized geological strength indices (GSI) and weathering grades classified the rock mass as highly fragmented in outcrop areas. This fragmentation is manifested as open joints, rock discoloration, and granular disintegration, significantly reducing the intact rock strength and inter-block cohesion along discontinuities.

### Basis for laboratory and numerical modeling parameters

The parameter selection for the subsequent scaled shaking table tests and 3DEC discrete element simulations was directly derived from the field characterization data presented above to encapsulate the most representative and critical conditions:

1. Joint Inclination (β): The angles of 70°, 80°, and 90° were selected to represent the lower bound, mean, and upper bound of the measured dominant dip range (75°-90°), allowing investigation of the failure mode transition and sensitivity.
2. Hazardous Rock Size (b): The dimensions of 0.5 m, 1.0 m, and 1.5 m were chosen as canonical values spanning the characteristic field-measured size range (0.4–1.8 m), corresponding to scenarios of severe, moderate, and mild fragmentation, respectively.
3. Rock Mass Fragmentation/Weathering Degree: The variation in simulated block size directly represents the degree of fragmentation. Furthermore, the reduced mechanical properties (cohesion, friction angle) assigned to discontinuities in the numerical model account for the weathering effects observed in situ.

This direct linkage ensures that the mechanistic studies conducted in this research are grounded in realistic geological conditions, thereby enhancing the practical relevance and application potential of the findings for hazard assessment along the CPEC.

## 3DEC numerical simulation

The shaking table model test focuses on the fracture degree, height, joint angle and seismic strength of the hazardous rock but does not consider the effect of the shape (aspect ratio) of the hazardous rock. To improve the system, a critical parameter, the hazardous rock body shape (aspect ratio), was added to the numerical simulation in this study.3DEC exhibits powerful solid modeling functionality, capable of producing both regular and intricate mesh models to accommodate diverse simulation conditions, which`s the main reason for the application of this paper. A four-factor, three-level experimental design was adopted: joint inclination β (70°, 80°, and 90°), hazardous rock size b (0.5 m, 1.0 m, and 1.5 m, corresponding to severe, moderate, and mild fragmentation, respectively), aspect ratio δ (0.67, 1, and 1.5), and the number of layers n (5, 10, and 15 layers). As shown in Table 3,through 81 sets (3^4) of numerical simulation experiments^5,27,28^, which are shown in Table 5, the mechanism of the influence of each parameter on the destabilization mode of the hazardous rock under basic seismic action was systematically investigated, with a focus on the effect of the irregular morphology of the hazardous rock body due to joint cutting.

**Table 5:** Simulation scheme.

No	Sectional inclination/(°)	Hazardous rock dimensions/m	Hazardous rock shape (aspect ratio)	Number of hazardous rock layers
1–3	90	1.5	0.67	15, 10, 5
4–6	90	1.5	1	15, 10, 5
7–9	90	1.5	1.5	15, 10, 5
10–12	90	1	0.67	15, 10, 5
13–15	90	1	1	15, 10, 5
16–18	90	1	1.5	15, 10, 5
19–21	90	0.5	0.67	15, 10, 5
22–24	90	0.5	1	15, 10, 5
25–27	90	0.5	1.5	15, 10, 5
28–30	80	1.5	0.67	15, 10, 5
31–33	80	1.5	1	15, 10, 5
34–36	80	1.5	1.5	15, 10, 5
37–39	80	1	0.67	15, 10, 5
40–42	80	1	1	15, 10, 5
43–45	80	1	1.5	15, 10, 5
46–48	80	0.5	0.67	15, 10, 5
49–51	80	0.5	1	15, 10, 5
52–54	80	0.5	1.5	15, 10, 5
55–57	70	1.5	0.67	15, 10, 5
58–60	70	1.5	1	15, 10, 5
61–63	70	1.5	1.5	15, 10, 5
64–66	70	1	0.67	15, 10, 5
67–69	70	1	1	15, 10, 5
70–72	70	1	1.5	15, 10, 5
73–75	70	0.5	0.67	15, 10, 5
76–78	70	0.5	1	15, 10, 5
79–81	70	0.5	1.5	15, 10, 5

### destabilization damage model for hazardous rocks

**Fig. 7:**
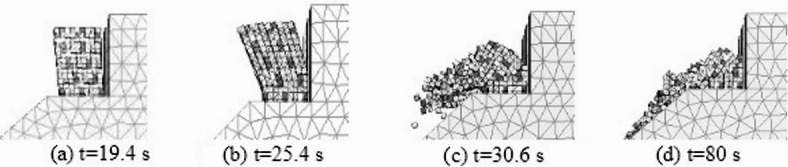
Destructive process of toppling collapse. (a) t = 19.4 s, (b) t = 25.4 s, (c) t = 30.6 s, (d) t = 80 s.

The four frames in Fig. 7 show the collapse damage of a steeply layered hazardous rock cliffs with seismic wave propagation at a joint inclination angle of 90°, a hazardous rock size of 1.0 m, a hazardous rock shape of 1, and 15 hazardous rock layers. The outermost and uppermost blocks of the hazardous rock body collapse earliest, and the motion process of deformation and destruction of the hazardous rock body can be roughly divided into three stages: fissure development, toppling and collapse, and stacking and stabilization. In this work, destabilization damage with this dynamic damage process is defined as toppling collapse.

**Fig. 8:**
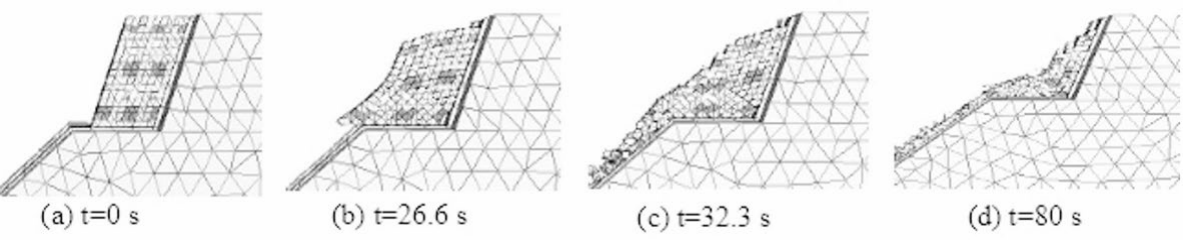
Destructive process of sliding collapse. (a) t = 0 s, (b) t = 26.6 s, (c) t = 32.3 s, (d) t = 80 s.

As shown in Fig. 8, during the propagation of horizontally oriented seismic waves, the hazardous rock mass tends to deform toward the free face, and the seismic force and gravity force of the upper hazardous rock cause the hazardous rock mass to slide along the steep cliff face. Slip occurs when the centre of gravity of the outermost and lowermost hazardous rock moves beyond the slope face, resulting in the loss of support of the upper rock mass, which displaces along the joints in the proclivity direction under the dominant force of gravity.Consider the structural plane characteristics and failure patterns of slope rock masses, a comparison of Figs. 6 and 7 reveals that the destabilization and damage modes of steep cliffs can be classified into two categories: toppling collapse and slipping collapse.These clearly defined failure modes, derived directly from the numerical simulations, establish a benchmark dataset. They will serve as the ground truth for training and validating a subsequent BP neural network model, which aims to predict failure modes based on key input parameters. The comparison between simulated failure modes and BP network predictions will be used to evaluate the reliability and applicability of the proposed predictive model.

### Study of the factors influencing the destabilization pattern of hazardous rocks

To study the effects of steeply layered hazardous rock cliff joint inclination, size, shape, and number of critical layers on different damage modes of hazardous rock bodies, the results of the numerical simulation are first classified into three categories according to the damage modes of toppling collapse damage and slipping collapse: stable, toppling collapse, and slipping collapse.

**Fig. 9:**
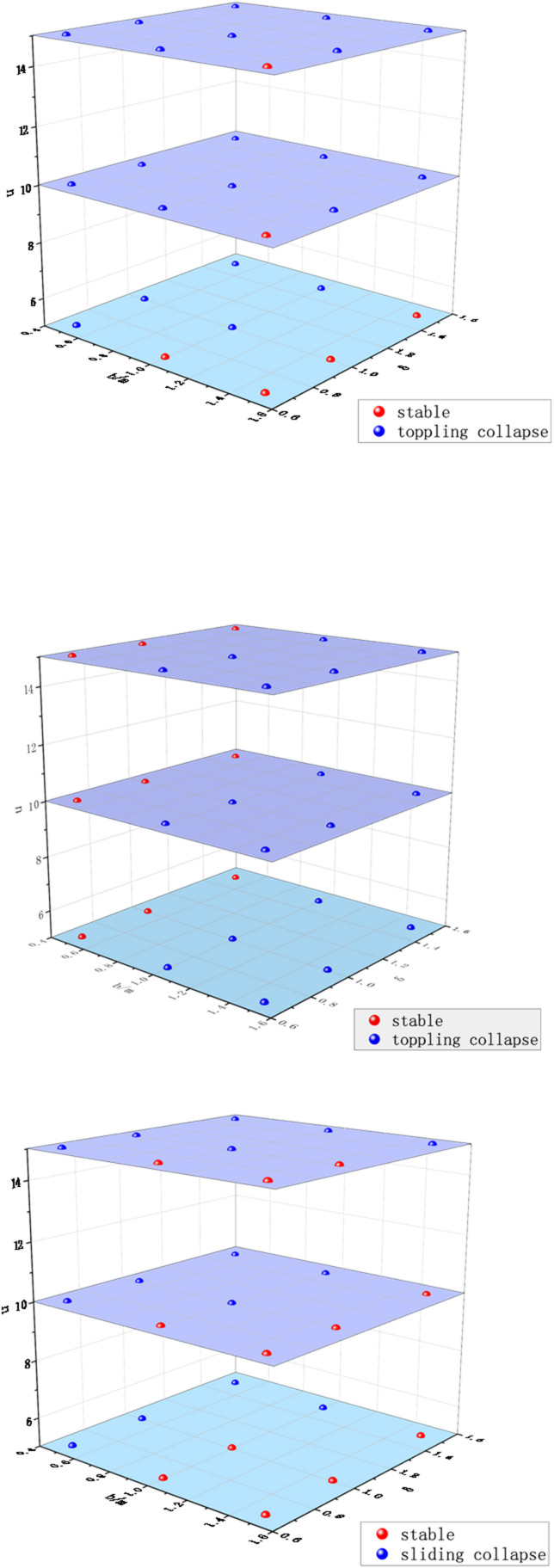
Three-dimensional distribution of hazardous rock failure patterns under different conditions.

The influence of key parameters on failure modes is comprehensively visualized in Fig. 9, which presents the three-dimensional distribution of simulation outcomes (stable, toppling collapse, and slipping collapse) across the 81 designed scenarios. A statistical summary of these results reveals a total of 27 stable cases, 15 slipping collapses, and 39 toppling collapses, indicating that toppling is the predominant failure mode within the studied parameter space and thus warrants focused investigation. Consistent with this finding, the joint inclination is confirmed as the primary controlling factor for the destabilization mode: when the inclination is 80–90°, the hazardous rock body predominantly exhibits toppling collapse, whereas a decrease to 70° shifts the dominant mode to slipping collapse. However, Fig. 9 also illustrates that parameters such as the block size, shape, and number of layers significantly modulate the failure pattern and stability even for a given joint inclination, highlighting a complex nonlinear coupling among these factors. Given the difficulty in establishing an explicit mathematical relationship between these coupled parameters and the resulting damage pattern using traditional methods, this study employs a neural network approach to predict stability under multiparameter coupling, thereby providing more accurate technical support for early warning and geological hazard prevention.

## BP neural networks for predicting instability patterns of hazardous rock bodies

### BP neural network fundamentals and learning algorithms

The back propagation (BP) neural network, as a typical feedforward artificial neural network, has a powerful nonlinear mapping capability.It not only possess general computational capabilities for processing numerical data, but also exhibit cognitive, learning, and memory capabilities for processing knowledge. They employ a method akin to a “black box,” utilizing learning and memory to identify nonlinear relationships (mappings) between input and output variables. When addressing problems and seeking solutions, the data obtained is input into the pre-trained network, which then performs network inference based on the knowledge it has acquired, thereby deriving reasonable answers and results.

**Fig. 10:**
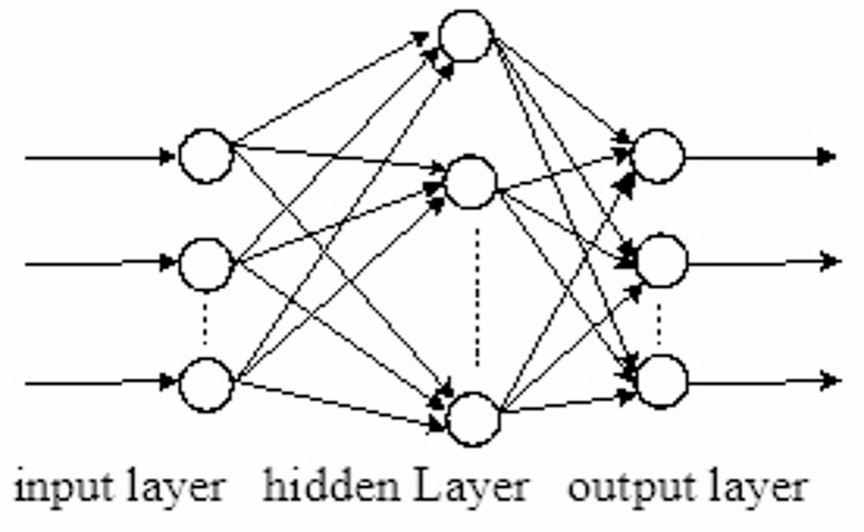
Schematic diagram of the BP network.

This paper focuses on its unique back propagation algorithm, which makes it possible to accurately construct complex mapping models through autonomous learning without pre-establishing the mathematical relationship between inputs and outputs. As shown in Fig. 10, the network adopts a hierarchical topology consisting of input, hidden and output layers. This structural feature makes it highly applicable in the fields of geotechnical engineering stability analysis, image recognition, economic prediction, etc., which is especially suitable for solving complex nonlinear problems such as the prediction of the instability patterns of hazardous rocks^29^ studied in this paper.

**Fig. 11:**
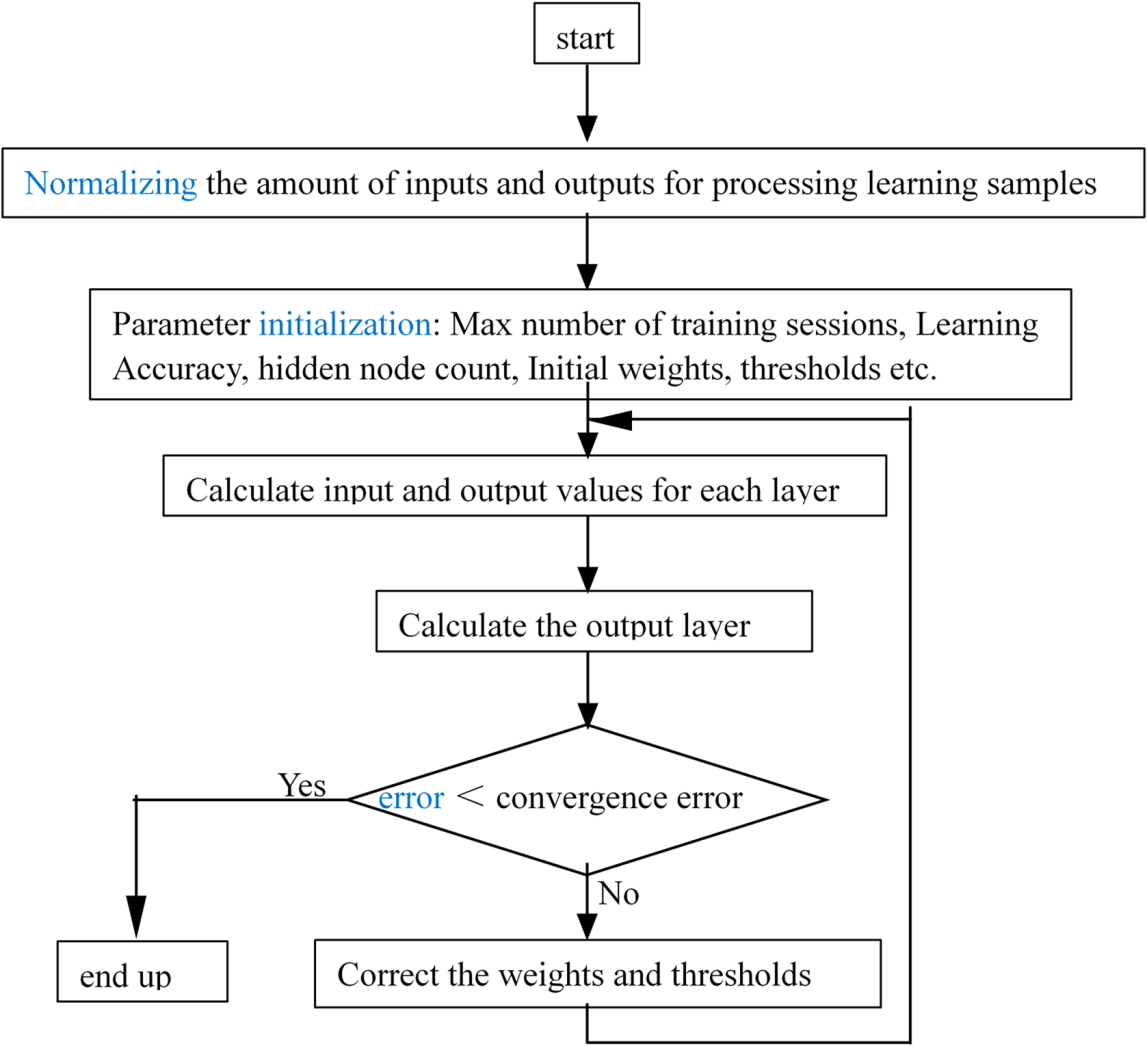
Flowchart of the BP algorithm procedure.

Sample learning is carried out using the steepest descent method. To minimise the sum of squared errors in the neural network, the weights and thresholds must be continuously adjusted during back propagation.First, learn the input and output values of the sample. After learning, enter the initialization and perform calculations. Finally, determine whether to end after judging the output value error.( the steps are detailed in Fig. 11).

The BP algorithm is grounded in solid theory, features a rigorous derivation process, achieves high accuracy, and generalizes well. However, it also has disadvantages. including slow convergence, susceptibility to local minima, and difficulty in determining the number of hidden layers and the number of nodes in the hidden layer. To address the above drawbacks, this study employs a Levenberg‒Marquardt (L-M)-based BP neural network to predict the instability mode of steeply layered hazardous rock bodies triggered by strong earthquakes. The weight update is carried out using the optimization expression shown in Eq. (1).

1 $$\Delta w = \left( {J^{T} J + \mu I} \right)^{{ - 1}} \cdot J^{T} e$$

where *e* is the error vector, *J* is the Jacobian matrix of the network error to the derivatives of the weights, *µ* is the adaptive factor, and *I* is the unit matrix.

### BP network structure for predicting destabilization patterns of hazardous rock bodies

**Fig. 12:**
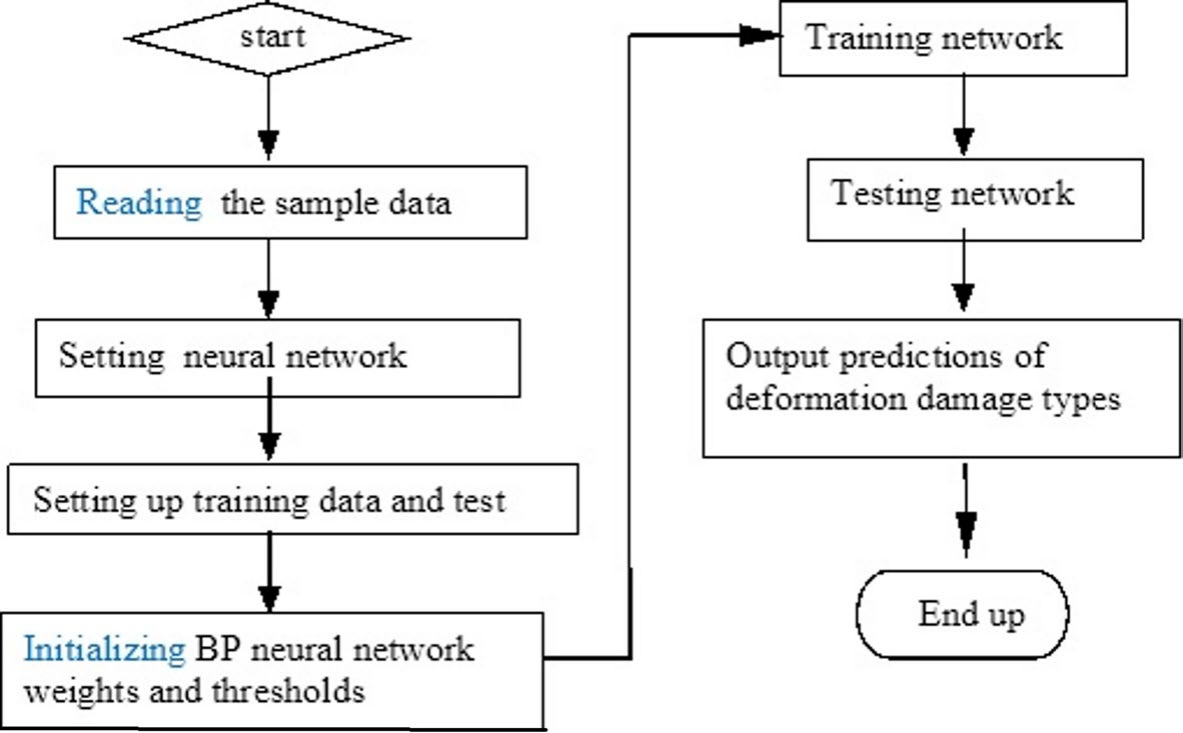
Flowchart of the BP neural network for predicting deformation damage types.

The BP neural network model constructed in this study uses the joint inclination angle, hazardous rock size, shape and number of layers as input features and the state of hazardous rock collapse as the output response. As shown in Fig. 12, through the systematic network training process, the model is able to autonomously learn the complex nonlinear relationship between each parameter and the instability mode. The trained prediction model can achieve the following functions: when new geological feature parameters of the hazardous rock body are input, it can quickly and accurately output its stability assessment results and possible destabilization modes, which can provide decision support for engineering practice. The modelling method overcomes the limitations of traditional analysis methods and significantly improves the accuracy and efficiency of hazardous rock stability prediction.

**Fig. 13:**
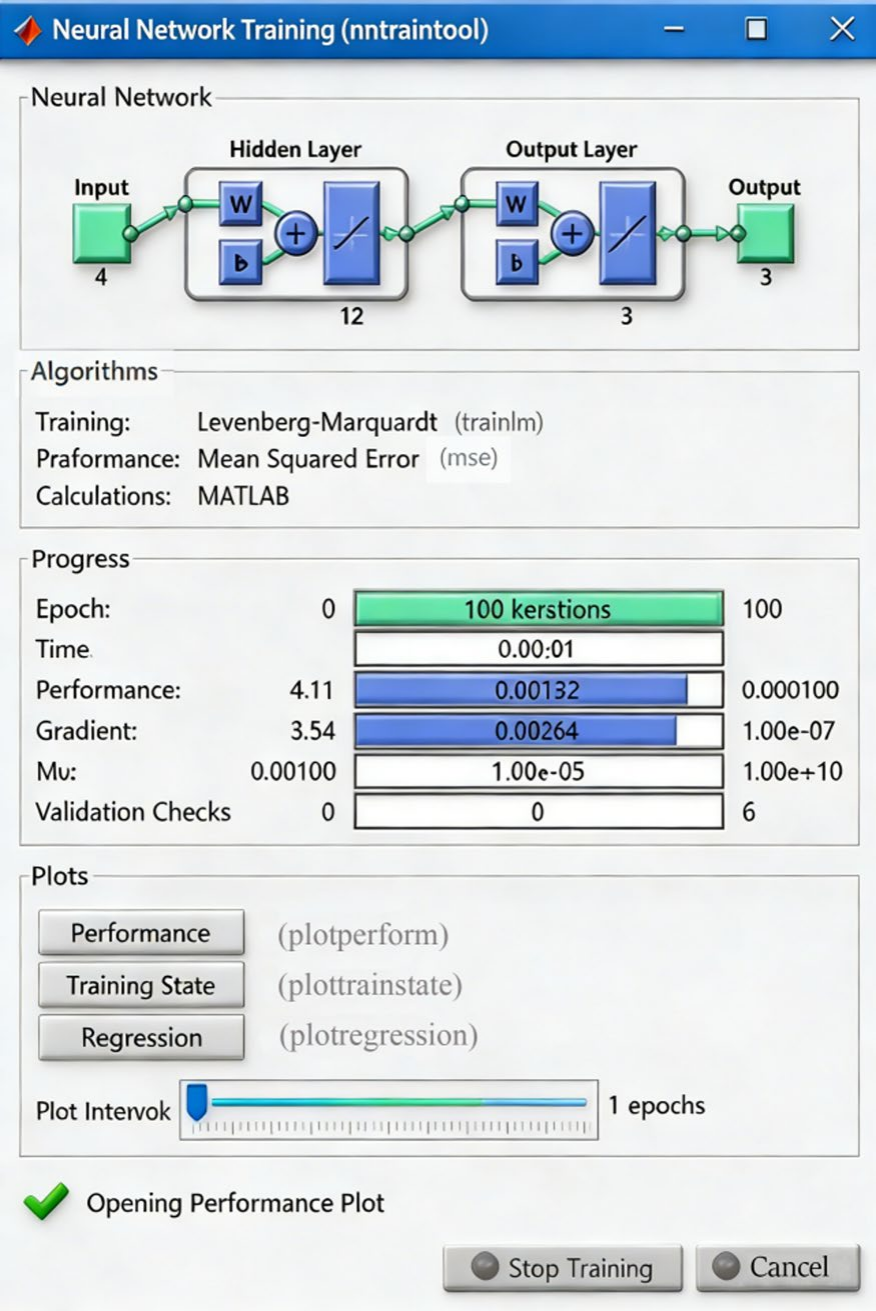
Diagram of the structure and parameters of the BP neural network.

According to Kolmogorov’s theorem, a three-layer network can approximate any function given sufficient training. On the basis of this principle, a 4-12-3 neural network structure is adopted (Fig. 13).

The input layer consists of 4 nodes, each representing one of the 4 influencing factors. The output layer includes 3 nodes, with 1, 2, and 3 representing the states of stable, tipping collapse, and sliding collapse, respectively, of 3 types of hazardous rocks. To determine the number of nodes in the hidden layer, a small number of nodes are preset to train the network and test the training error. The number of nodes is then gradually increased until the training error tends to stabilise. This approach follows a network structure growth type method.

**Fig. 14:**
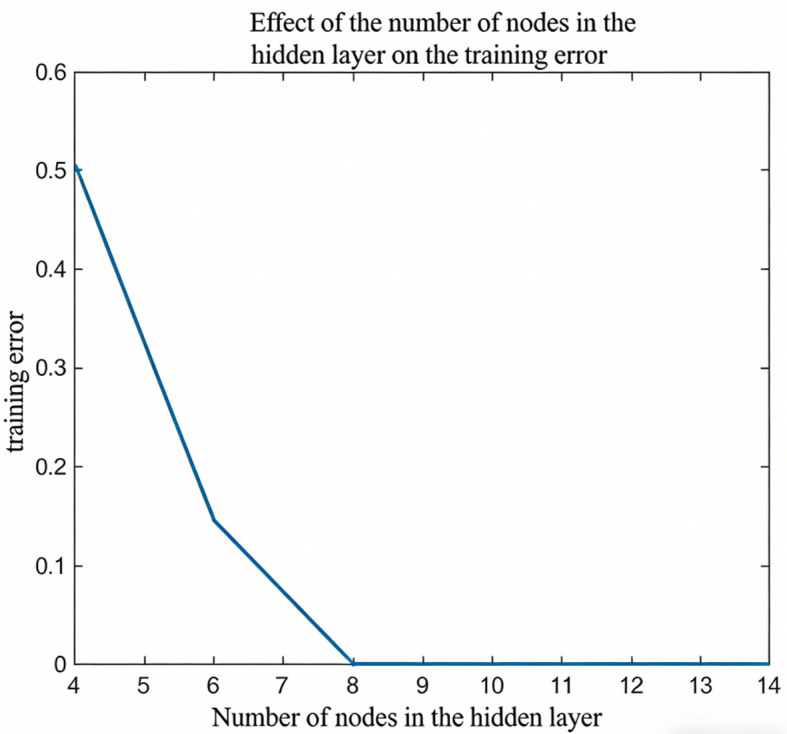
Effect of the number of nodes in the hidden layer on the training error.

**Fig. 15:**
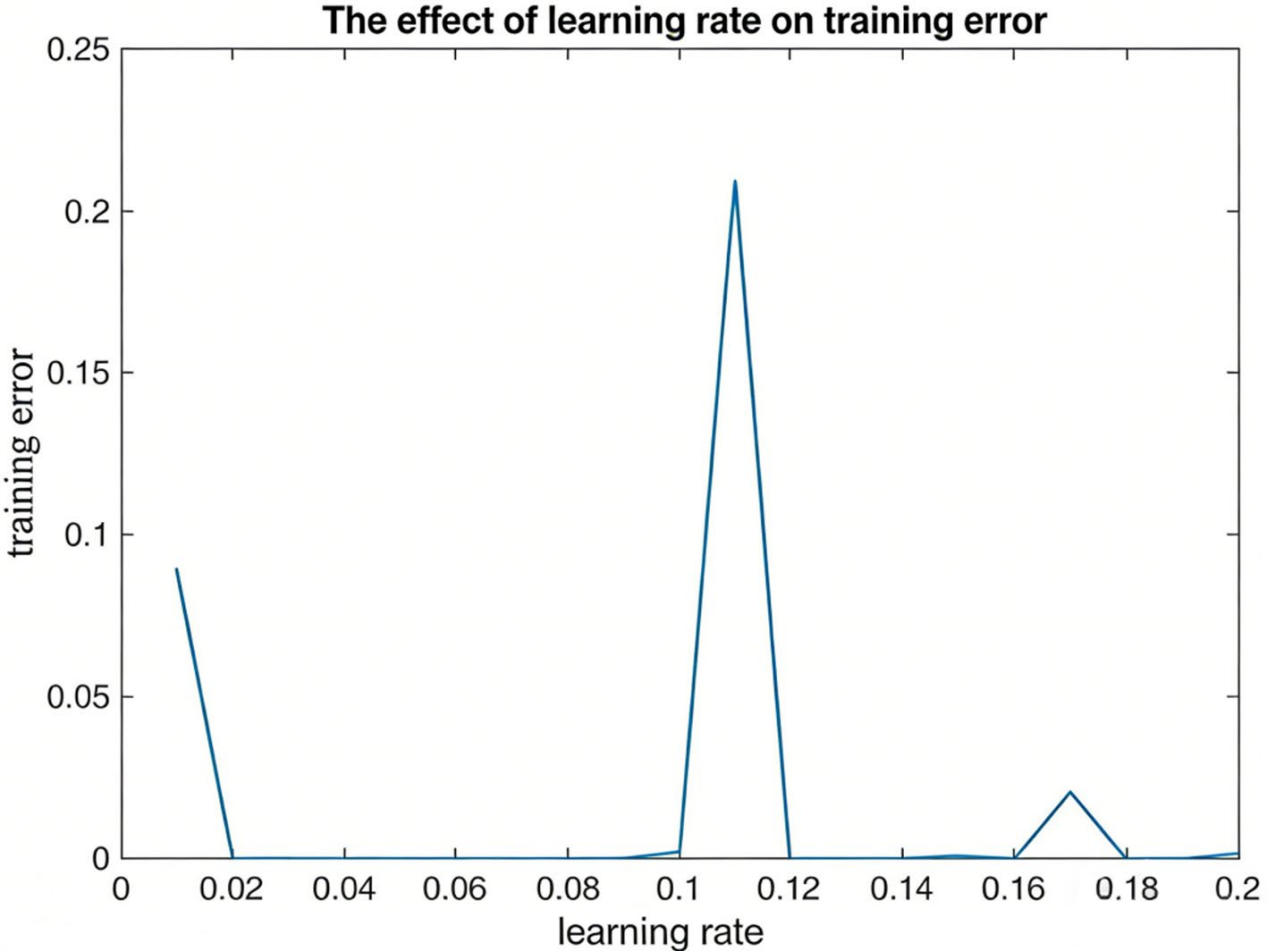
Effect of the learning rate on the training error.

As shown in Fig. 14, when the implied layer starts at 8, the training error is no longer significantly reduced. Therefore, it is determined that, the neural network is developed using MATLAB. The training set (66 groups) and test set (numbered 74, 16, 75, 20, 51, 70, 48, 57, 1, 13, 32, 53, 79, 7, and 69, for a total of 15 groups) are constructed through a scientific sample division method(Randomly selected). In terms of network structure design, the tansig transfer function is applied from the input layer to the implicit layer to capture the nonlinear features among the parameters, whereas the purelin linear function is used in the output layer to ensure the continuity of the prediction results. Among the key training parameters, the learning rate of the L-M algorithm is optimised to 0.07 (Fig. 15), which is a setting that effectively balancing the convergence speed of the model and the training accuracy, laying a solid foundation for the subsequent prediction performance analysis. Therefore, the optimal learning rate l_r_ is set to 0.07, the maximum number of training steps is set to 100, the mean square error (MSE) of the network output is the target, the goal is set to le-6, and the network was trained using the Levenberg-Marquardt backpropagation algorithm, which implements the L-M algorithm.

**Fig. 16:**
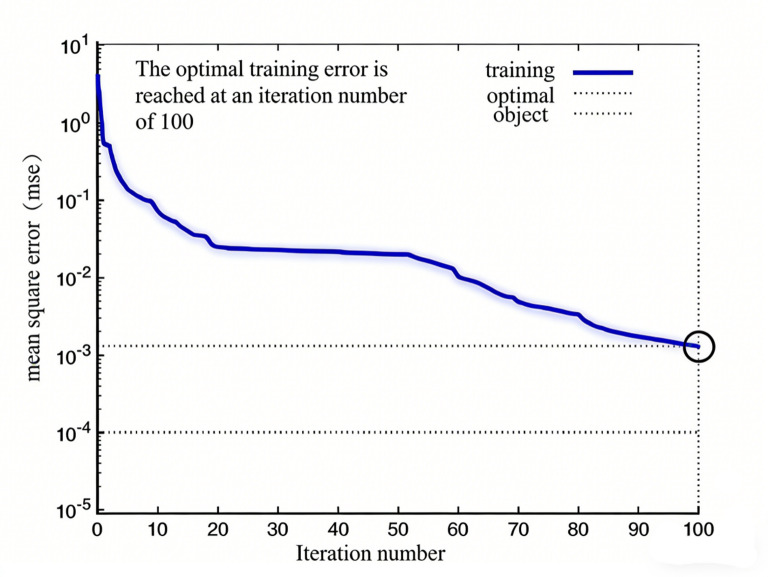
Convergence plot of the training error.

The error variation curve of the L-M algorithm is shown in Fig. 16. As can be seen from Fig. 16, after 100 training steps, the mean square error (MSE) of the network reached the target error criterion of 0.0013153. At this point, the BP neural network model for predicting the deformation and instability patterns of hazardous rockes was successfully established.

### Predictive performance analysis and comparison

To ensure the robustness of the model, the dataset was randomly split into training (66 samples) and testing (15 samples) sets. Additionally, a 5-fold cross-validation was performed on the training set, yielding an average accuracy of 85.2 ± 2.1%, confirming the model’s stability and generalizability.It is essential to assess the reliability of the BP neural network model for predicting the deformation and instability patterns of hazardous rock masses.Therefore, by inputting the predicted sample values(15 groups) into the model, corresponding prediction outcomes are generated. These analytical results are presented in Table 6.

**Table 6:** Analysis of the projected results.

No	Test sample	Sectional inclination/(°)	Hazardous rock dimensions/m	Hazardous rock shape (aspect ratio)	Number of hazardous rock layers	Projected results	Actual result
74	1	70	0.5	0.67	10	3	3
16	2	90	1	1.5	15	2	2
75	3	70	0.5	0.67	10	3	3
20	4	90	0.5	0.67	10	2	2
51	5	80	0.5	1	5	2	2
70	6	70	1	1	5	3	3
48	7	80	0.5	0.67	5	2	2
57	8	70	1.5	0.67	5	1	1
1	9	90	1.5	0.67	15	2	1
13	10	90	1	1	15	2	2
32	11	80	1.5	1	10	1	1
53	12	80	0.5	1.5	10	2	2
78	13	70	0.5	1.5	15	3	3
7	14	90	1.5	1.5	15	2	2
69	15	70	1	1	5	3	1

**Table 7:** Confusion matrix results.

Actual \ Predicted	Stable	Toppling	Sliding
Stable	4	1	0
Toppling	1	6	0
Sliding	0	0	3

**Fig. 17:**
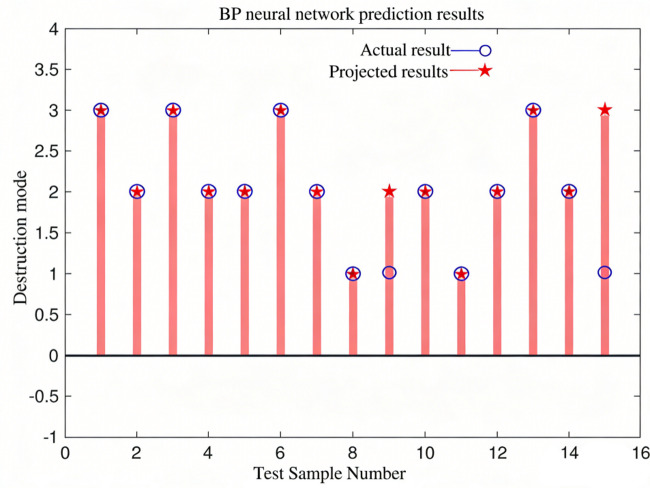
Results of the BP neural network prediction.

Analysis of the prediction results in Table 6; Fig. 17 shows that, except for errors of serial numbers 9 and 15, the remaining test samples are correctly predicted, resulting in a prediction accuracy of 86.7%. In addition to overall accuracy, a confusion matrix (Table 7) was constructed to evaluate the classification performance of the BP neural network model across the three failure modes: stable (Class 1), toppling collapse (Class 2), and sliding collapse (Class 3). The matrix demonstrates that the model performs well in distinguishing between toppling and sliding collapse, with only two misclassifications occurring between stable and toppling collapse. The predicted and actual results are compared by plotting the test sample number on the horizontal axis and the deformation damage pattern on the vertical axis. The results predicted by the BP neural network are shown in Fig. 17.

To further quantify the predictive performance of the BP neural network, the coefficient of determination (R²) and root mean square error (RMSE) were calculated for the regression output of critical collapse vibration velocity. The model achieved an R² of 0.92 and an RMSE of 0.045 m/s on the test set, indicating strong predictive capability and low error magnitude.

## Study on the critical state of hazardous rock collapse

Considering that the China-Pakistan Highway section (within the country) has experienced landslides and that the results of the 3DEC numerical simulation tests are mainly dominated by toppling landslides, the next step of this paper is to conduct further research on the toppling landslide model.In the preceding section of this paper, the displacement angle method was introduced through vibration table tests to predict the displacement angle threshold prior to the collapse of unstable rock masses. The limitations of vibration table tests were supplemented by 3DEC discrete element numerical simulations, which classified the collapse patterns of unstable rock masses. A BP neural network was used to construct a predictive model for the instability patterns of unstable rock masses. Therefore, it is necessary to introduce physical indicators for rapidly determining whether unstable rock masses are in a critical collapse state.

In this work, we use 3DEC software to simulate hazardous rock collapse under strong earthquakes. We study and analyse the influence of four major factors, joint inclination, hazardous rock size, hazardous rock shape, and number of hazardous rock layers, on the characteristics of collapse damage. We aim to determine the physical indicators and thresholds for identifying hazardous rocks at the critical collapse stage. With support from a BP neural network, we aim to identify the main risk factors influencing collapse damage of hazardous rocks to provide a basis for further research on the assessment of collapse disasters. These findings provide a basic basis for further research on collapse disaster assessment.

### Method for determining the speed of a hazardous rock collapse

**Fig. 18:**
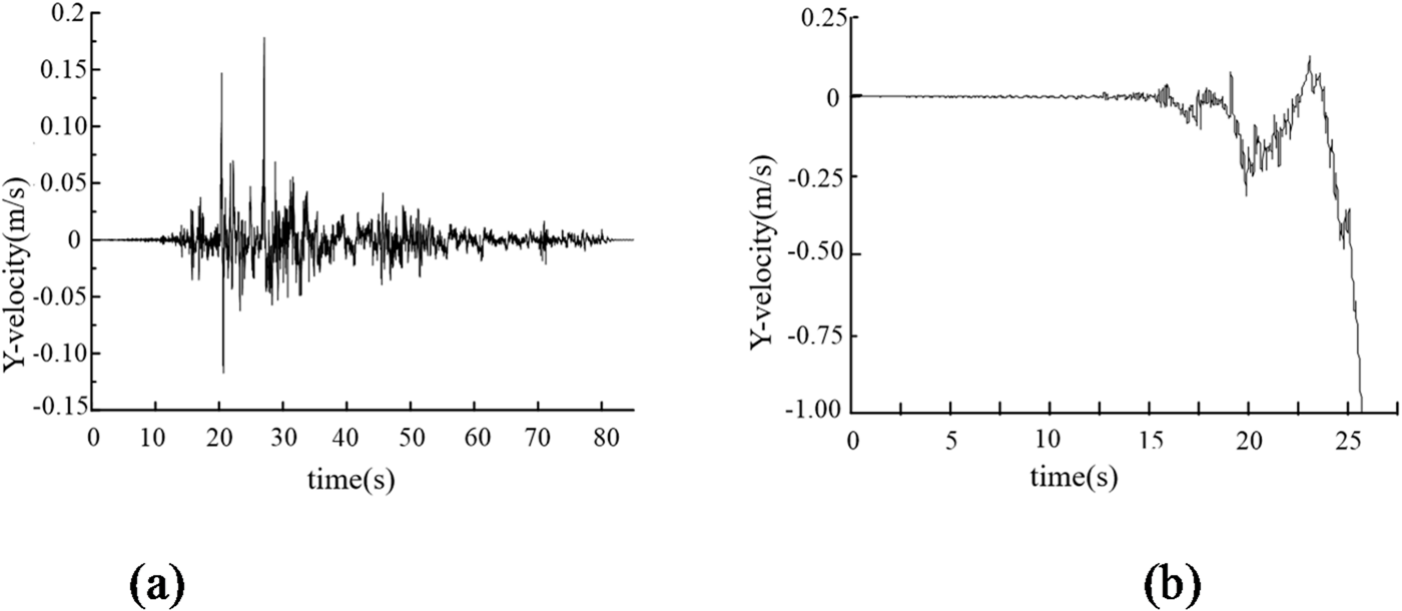
Vertical velocity curves of non-collapsed and collapsed blocks. (a) Non-collapsed, (b) Collapsed.

To accurately determine the onset of collapse in steep cliff hazardous rocks, time-course curves of the displacement, velocity, and interblock stress in the outermost and uppermost blocks of the hazardous rock body during collapse are monitored. A comparison reveals that the velocity time-course curve changes significantly, whereas the time-course curves of other physicomechanical indicators cannot be determined. As the upper block loses support owing to accumulated lateral displacement, stability decreases until collapse occurs. Dates were monitored by 3DEC software and vertical velocity curves (Y-direction, vertically upwards is positive) are chosen to compare the velocities of noncollapsed and collapsed blocks, as shown in Fig. 18.

To quantitatively and objectively identify this critical point, a method based on the fundamental mathematical definition of an inflection point was employed. The vertical displacement-time (Y-t) data obtained from 3DEC monitoring were exported and analyzed using Origin software. The critical collapse initiation time was rigorously defined as the point where the second derivative of the Y-t curve with respect to time equals zero: d²Y/dt² = 0 (1). The specific operational steps in Origin were as follows: (1) Plot the Y-t data and set the curve as the active graph; (2) Apply the ‘Analysis’ → ‘Mathematics’ → ‘Differentiate’ tool; (3) In the differentiation settings, set the ‘Order’ to ‘2’ to compute the second derivative; (4) Output the resulting second derivative curve to a new worksheet; (5) Identify the position where the second derivative values cross zero as the inflection point. The time corresponding to this point is defined as the collapse time. This point also coincides with the maximum value of the first derivative (vertical velocity), marking the peak descent speed attained just before the block transitions into unrestrained free-fall.

This inflection point (d^2^Y/dt^2^ = 0) carries profound physical significance: it marks the instant when the vertical velocity peaks immediately before the block enters free-fall. This peak in kinetic energy coincides with the sudden release of stored strain energy from the rock mass and the instantaneous loss of vertical constraint, causing the vertical acceleration to drop to zero. Therefore, this mathematically defined criterion precisely captures the transition from constrained rocking to irreversible collapse, representing the onset of gravitational free-fall.

Consequently, in this work, the vertical velocity at this moment is defined as the critical collapse vibration velocity, and the corresponding moment of the critical collapse vibration velocity is defined as the point in time when the block begins to separate from the surrounding rock mass. This velocity judgment method is used to determine the onset of collapse damage under different influencing factors and to analyze the influence of the aforementioned four factors on the collapse initiation.

Figure 18(b) illustrates the vertical velocity-time curve for a collapsed block. According to the mathematical criterion established in the preceding paragraph (d²Y/dt² = 0), the precise moment of collapse initiation is objectively identified. Before this inflection point, the vertical velocity behavior is similar to that of the non-collapsed block shown in Fig. 18(a), characterized by fluctuations within a bounded range. After the inflection point, the curve exhibits a sharp, nearly linear increase, indicating that the block has entered a state of accelerating downward movement—a kinematic signature of irreversible collapse, coinciding with the loss of support as its center of gravity moves beyond the slope face.

Based on this objective definition, the vertical velocity at the moment corresponding to the inflection point (d²Y/dt² = 0) is termed the critical collapse vibration velocity. The velocity judgment method, grounded in this mathematical criterion, is subsequently applied to determine the onset of collapse under the influence of different factors and to analyze how the four primary factors affect this critical timing.

**Fig. 19:**
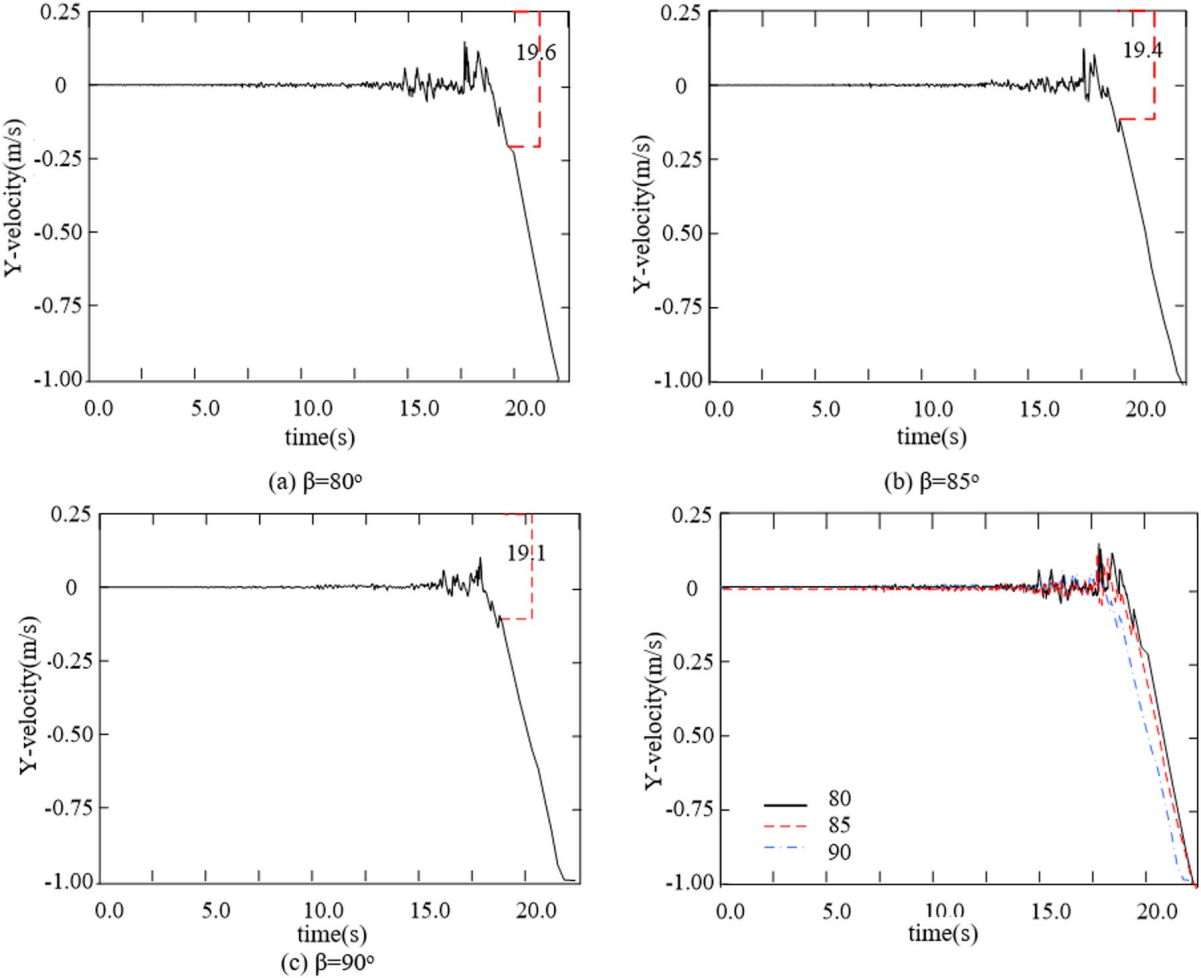
Vertical velocity time course diagram of the joint inclination change.

**Fig. 20:**
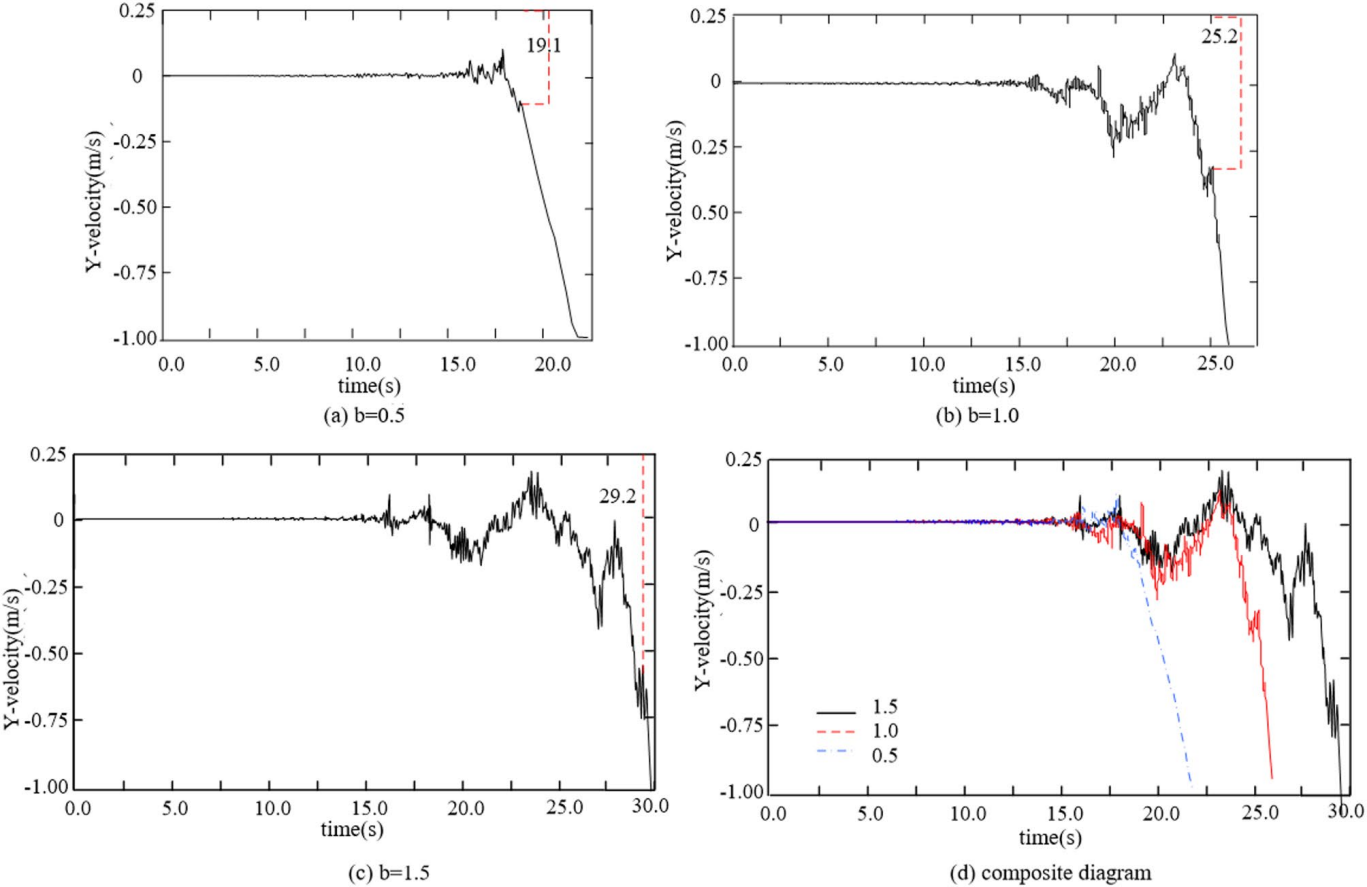
Time course plot of the vertical velocity with respect to the size of hazardous rock.

**Fig. 21:**
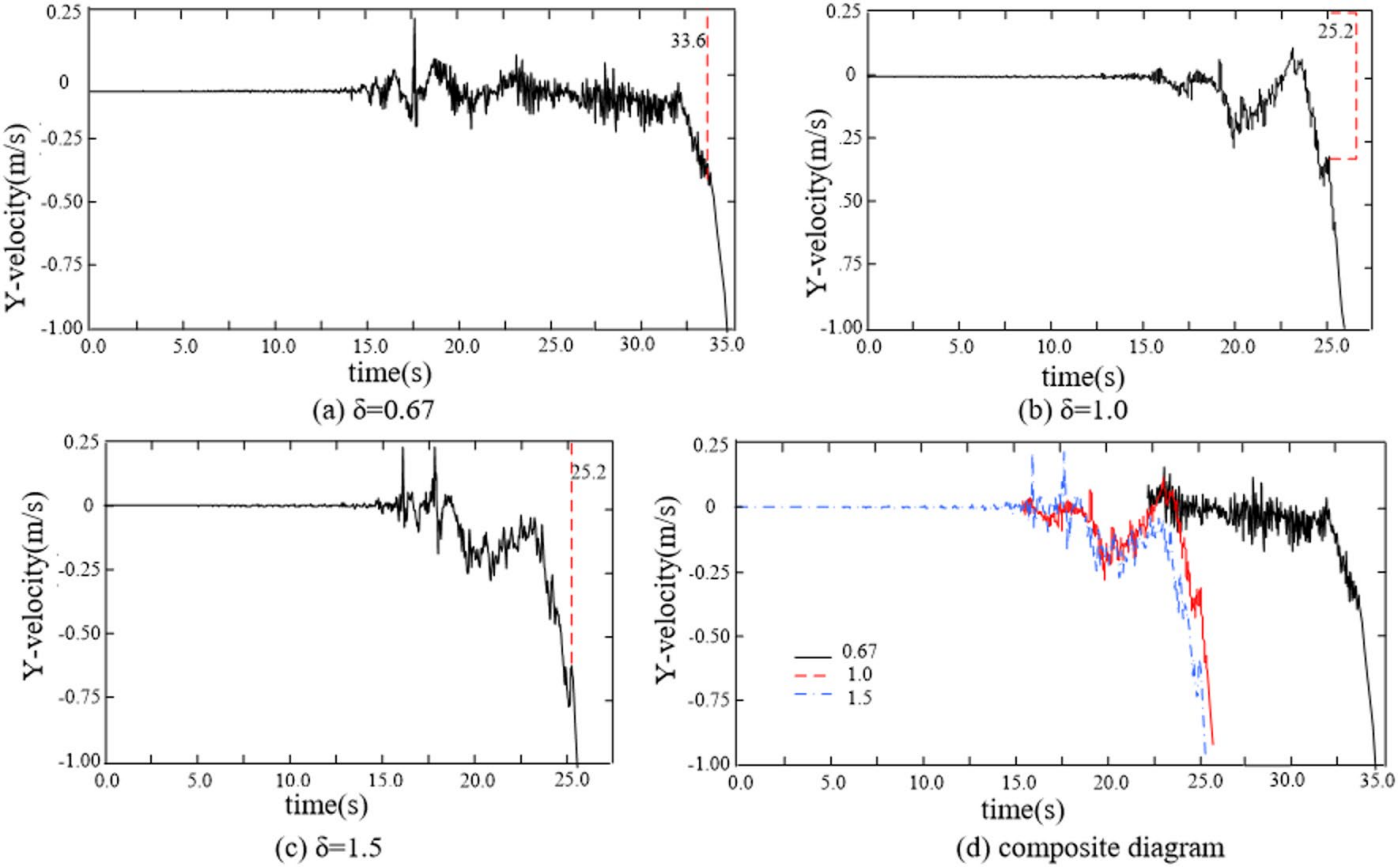
Time-course plot of the vertical velocity for the shape change of hazardous rock.

**Fig. 22:**
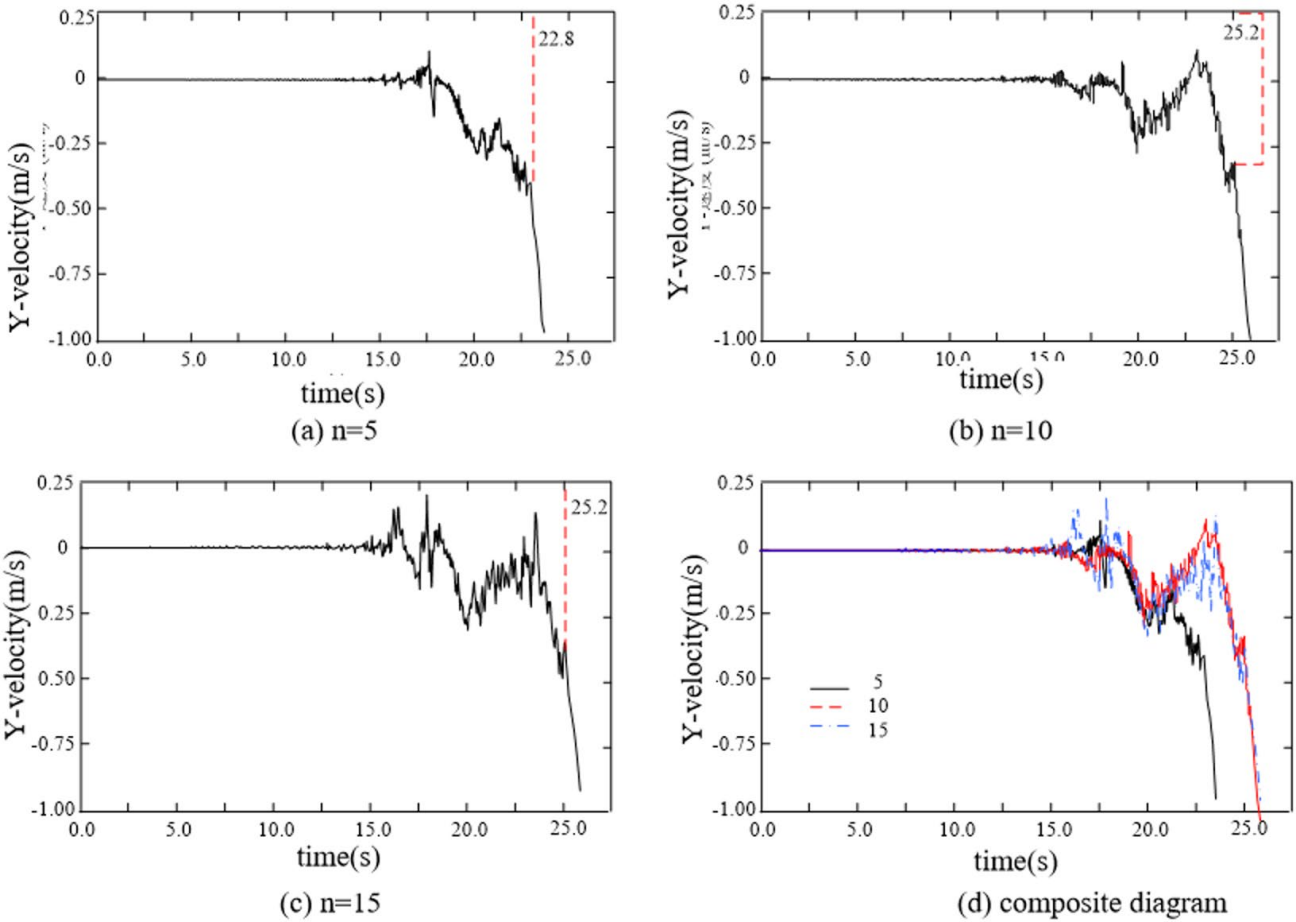
Time-course plot of the vertical velocity with respect to the number of hazardous rock layers.

A simulation with a joint inclination of 70° revealed a destabilization pattern distinctly different from the toppling collapse shown in Fig. 7, more closely resembling a sliding failure where the outermost and lowest blocks initiate movement. Therefore, scenarios with a 70° joint inclination were excluded from the subsequent analysis focused on the timing of toppling collapse. Using the vertical velocity data of the outermost and uppermost blocks from the 3DEC simulations, time-course curves were plotted for scenarios with varying joint inclinations. To facilitate comparison of the effect of joint inclination on the collapse onset, the vertical velocity time courses for three representative scenarios are compiled in a single graph, as shown in Figs. 19, 20, 21 and 22.

Figure 19 shows that when the joint inclination angle is 80°, the vertical velocity curve changes abruptly at 19.6 s, the vertical velocity fluctuates between 0 and 19.6 s, the hazardous rock body is in a self-stabilising state, the vertical velocity of the block grows substantially and rapidly after 19.6 s, and the block accelerates downwards movement. Therefore, the hazardous rock body collapses at 19.6 s. Similarly, when the joint inclination angles are 85° and 90°, the time points at which the hazardous rock body initiates collapse damage are 19.4 s and 19.1 s, respectively. The composite diagram in Fig. 18 shows that when the joint inclination angles are 80°, 85°, and 90°, the time of collapse damage initiation increases with increasing joint inclination angle, and the hazardous rock body is more prone to collapse. However, the time advance is not too large, and collapse occurs at approximately 19.4 s. When the joint inclination angle is 70°, the hazardous rock body is destabilised in the mode of slipping collapse, which indicates that the joint inclination angle has less influence on the onset moment of collapse damage of the hazardous rock body of small size and a more critical influence on the mode of damage of the steep cliff laminar hazardous rock.

Figure 20 shows that when the size of the hazardous rock is 0.5 m, the vertical velocity curve changes abruptly at 19.1 s, the vertical velocity fluctuates in a certain range from 0 to 19.1 s, and the hazardous rock body is in a self-stabilising state. After 19.1 s, the vertical velocity of the block grows substantially and rapidly, and the downwards movement of the block accelerates. Therefore, the hazardous rock body collapses at 19.1 s. Similarly, when the sizes of the hazardous rock are 1.0 m and 1.5 m, the onset of the collapse damage of the hazardous rock body occurs at 25.2 s and 29.2 s, respectively. The composite diagram in Fig. 19 shows that the onset of the collapse damage of the hazardous rock body advances with the reduction in the size of the hazardous rock. This finding indicates that the more broken the hazardous rock body is, the more rapid and easier the collapse. Therefore, the hazardous rock size and overall stability of the hazardous rock are positively correlated. When the hazardous rock size ranges from 0.5 m to 1.5 m, the collapse onset moment is delayed by 10.1 s, which indicates that the hazardous rock size has a great influence on the stability of the hazardous rock body. Therefore, the degree of fragmentation is first observed when the stability of the laminated hazardous rock body is determined.

Figure 21 shows that when the shape value of the hazardous rock is 0.67, the vertical velocity curve changes abruptly at 33.6 s, the vertical velocity fluctuates in a certain range from 0 to 33.6 s, and the hazardous rock body is in a self-stabilising state. After 33.6 s, the vertical velocity of the block grows substantially and rapidly, and the block accelerates downwards. Therefore, the hazardous rock body can be considered to have collapsed at 33.6 s. Similarly, when the hazardous rock shape values are 1.0 and 1.5, the onset of collapse damage of the hazardous rock body occurs at 25.2 s. The composite graph in Fig. 20 shows that the onset of collapse damage of the hazardous rock body tends to decrease and then remains unchanged with increasing hazardous rock shape. When the hazardous rock shape δ value is less than 1, the onset moment of collapse damage increases with increasing hazardous rock shape value, and the two parameters are negatively correlated. When the hazardous rock shape δ value is greater than 1, increasing the aspect ratio at this time does not affect the onset of the collapse of the hazardous rock, and the time point of the collapse overlaps. This finding suggests that there exists a threshold value of the onset moment of the collapse with the change in the hazardous rock shape.

Figure 22 shows that when the number of hazardous rock layers is 5, the vertical velocity curve changes abruptly at 22.8 s, the vertical velocity fluctuates within a certain range from 0 to 22.8 s, and the hazardous rock body is in a self-stabilising state. After 22.8 s, the vertical velocity of the block grows substantially and rapidly, and the block accelerates downwards. Therefore, the hazardous rock body can be considered to have collapsed at 22.8 s. Similarly, when the numbers of hazardous rock layers are 10 and 15, the onset time of the collapse damage of the hazardous rock body is 25.2 s. According to the composite diagram in Figs. 4 and 5, the onset time of the collapse damage of the hazardous rock body tends to increase and then remains unchanged with increasing number of hazardous rock layers. When the number of hazardous rock layers n is less than 10, the onset moment of collapse damage increases with increasing number of hazardous rock layers, and the two variables are positively correlated. The vertical velocity curves clearly separate. When the number of hazardous rock layers n is greater than 10, increasing the number of hazardous rock layers at this time does not affect the onset moment of the collapse damage to the hazardous rock layer, and the collapse moments basically overlap, which indicates that there exists a threshold value of the onset moment of the collapse with the change in the number of hazardous rock layers.

### Study of the critical collapse velocity of hazardous rock

The critical collapse vibration speed is analysed to identify precursor characteristics or critical collapse vibration speed threshold values of tipping collapse. This analysis aids in determining whether a hazardous rock body exhibits signs of tipping collapse. The critical vibration speed corresponding to the collapse mode depicted in Fig. 9 is determined using a velocity discrimination method for hazardous rock tipping collapse damage, and the results are shown in Table 8.

**Table 8:** Critical collapse velocity.

No	Sectional inclination/(°)	Hazardous rock dimensions/m	Hazardous rock shape (aspect ratio)	Number of hazardous rock layers	Critical collapse velocity/(m/s)
4	90	1.5	1	15	-0.7077
5	90	1.5	1	10	-0.8167
7	90	1.5	1.5	15	-0.3920
8	90	1.5	1.5	10	-0.5872
10	90	1	0.67	15	-0.3729
11	90	1	0.67	10	-0.4141
13	90	1	1	15	-0.5227
14	90	1	1	10	-0.4345
15	90	1	1	5	-0.3954
16	90	1	1.5	15	-0.4277
17	90	1	1.5	10	-0.5896
18	90	1	1.5	5	-0.5911
19	90	0.5	0.67	15	-0.4120
20	90	0.5	0.67	10	-0.1120
21	90	0.5	0.67	5	-0.4746
22	90	0.5	1	15	-0.3601
23	90	0.5	1	10	-0.1908
24	90	0.5	1	5	-0.1038
25	90	0.5	1.5	15	-0.2735
26	90	0.5	1.5	10	-0.2508
27	90	0.5	1.5	5	-0.1160
29	80	0.5	0.67	10	-0.4961
30	80	0.5	0.67	5	-0.0050
31	80	0.5	1	15	-0.1927
32	80	0.5	1	10	-0.2169
33	80	0.5	1	5	-0.1429
34	80	0.5	1.5	15	-0.3048
35	80	0.5	1.5	10	-0.1565
36	80	0.5	1.5	5	-0.0965

**Fig. 23:**
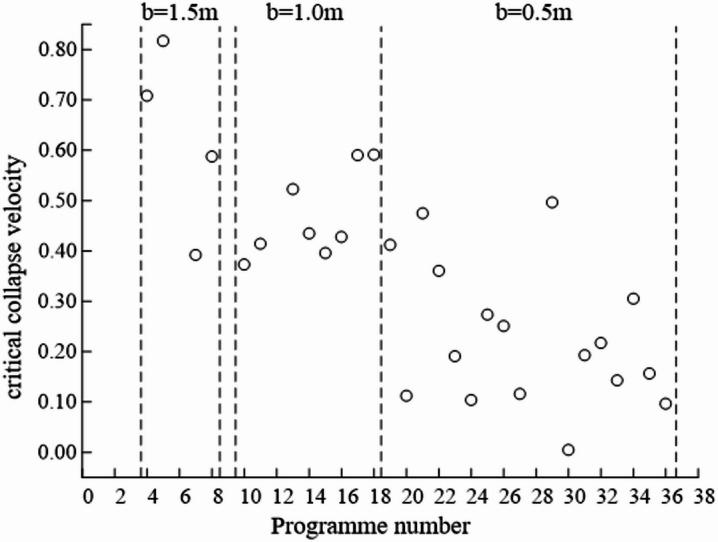
**A**bsolute value of the critical collapse velocity.

An analysis of the data in Table 5 reveals that the critical collapse velocities of precipitous rocks do not increase or decrease monotonically with increasing joint inclination, hazardous rock size, shape, or number of layers; instead, these velocities tend to fluctuate with no obvious pattern. However, the values of these critical collapse velocities are concentrated in an interval with a small fluctuation range under the same hazardous rock size, so it is assumed that the relationship between the critical collapse velocity of hazardous rocks and their size is quite close. Therefore, the critical collapse velocity of hazardous rocks is statistically classified by their size, and a normal distribution analysis is adopted to ensure that 90% of the critical velocities are within the boundaries to determine the upper and lower limits of the critical collapse velocities of hazardous rocks. The following section presents an analysis of the relationship between the absolute values of critical collapse velocities of hazardous rocks and their sizes, as shown in Fig. 23.

The figure shows that when the hazardous rock size is 1.5 m, the absolute value of the critical collapse vibration velocity is concentrated in the range of 0.58–0.80 m/s; when the hazardous rock size is 1.0 m, the absolute value of the critical collapse vibration velocity is concentrated in the range of 0.38–0.60 m/s; and when the hazardous rock size is 0.5 m, the absolute value of the critical collapse vibration velocity is concentrated in the range of 0.10–0.40 m/s. Through comprehensive analyses, although there is no correlation between the absolute value of the critical collapse velocity and the size of the hazardous rock, when the size of the hazardous rock is determined, other factors have almost no influence on the fluctuation range of the absolute value of the critical collapse velocity. The lower and upper limits of the absolute value of the critical collapse velocity of hazardous rock decrease with decreasing size, i.e., the smaller the size of the hazardous rock is, the lower the vibration velocity required to reach its critical collapse state, and the easier it is to collapse and destabilise the rock. Therefore, by monitoring the vertical velocity of hazardous rock, when the velocity is less than the lower limit of the critical collapse velocity, it can be assumed that the steeply layered hazardous rock cliffs have not tumbled, collapsed, and become unstable. When the monitored velocity is within the range of the critical collapse vibration velocity, an early warning prediction is made immediately, and corresponding preventive measures can be implemented. The critical collapse velocity as the size of the hazardous rock increases is shown in Table 9.

**Table 9:** Variation in critical collapse vibration velocity with hazardous rock size.

Hazardous rock dimensions/m	Critical collapse velocity/(m/s)
0.5	0.10 ~ 0.40
1.0	0.38 ~ 0.60
1.5	0.58 ~ 0.80

## Identification of risk factors for hazardous rock failure

**Fig. 24:**
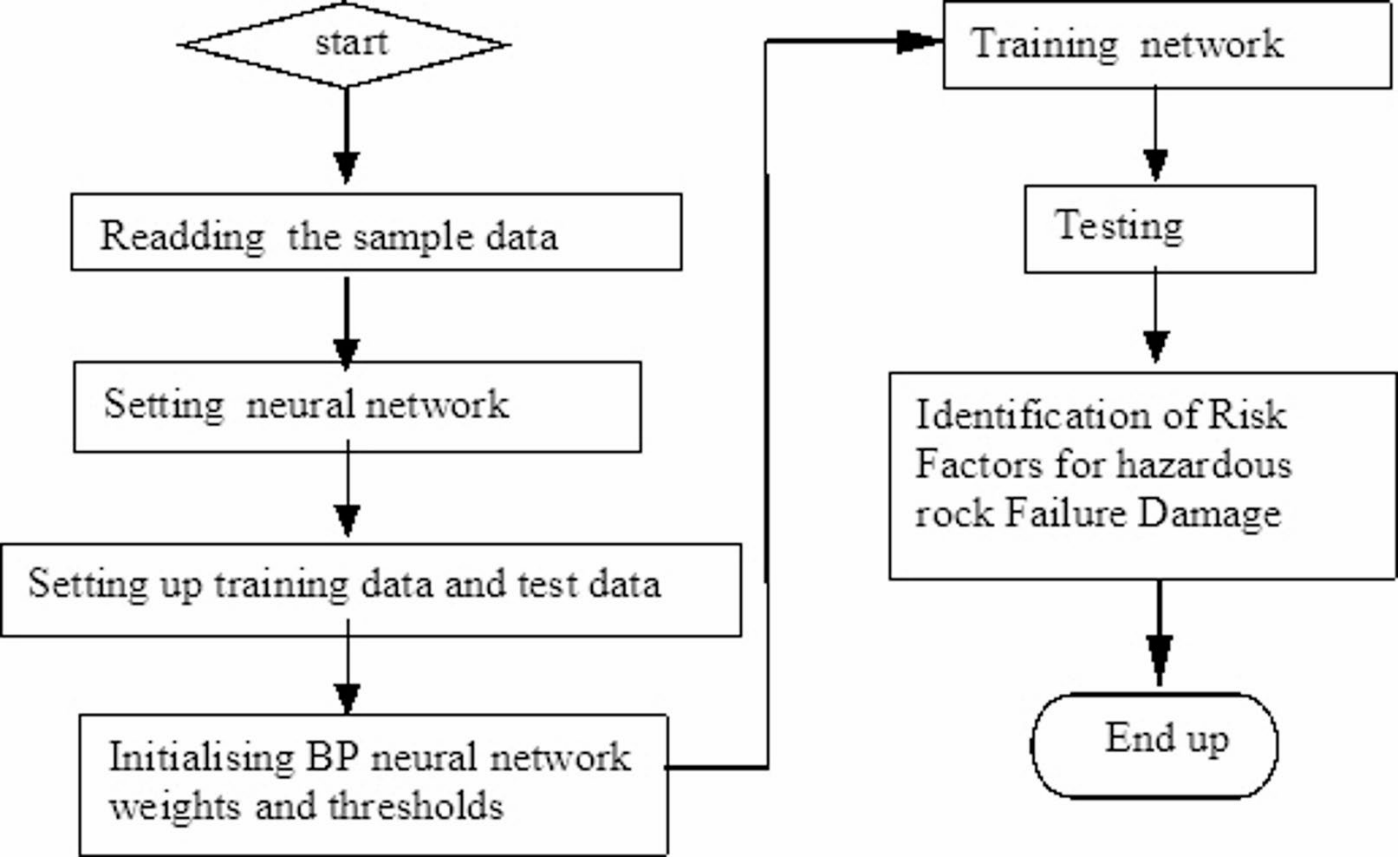
Risk factor identification process for BP neural network hazardous rock toppling collapse damage.

As mentioned in Sect.  5, factors such as the hazardous rock join inclination angle, size, shape, and number of dangerous layers significantly affect rockfall collapse damage, but the relative importance of each factor is not yet clear. This section aims to analyze the relative importance of each factor. A BP neural network is used to establish a risk factor model that can identify the toppling collapse damage of steep cliff-layered hazardous rocks. On the basis of the process shown in Fig. 24, once data on the tipping collapse of a hazardous rock body is obtained, the model predicts the critical collapse vibration speed value. Then, to determine the degree of influence of different risk factors, the mean absolute change in the critical collapse vibration speed is calculated under the premise that the input of research factor is increased by 2% while the remaining risk factors are unchanged. Consequently, appropriate and effective measures can be implemented for each risk factor to prevent the risk or minimize the resulting damage in a more targeted way.

### Prediction of critical collapse vibration speeds for layered hazardous rock toppling types

**Fig. 25:**
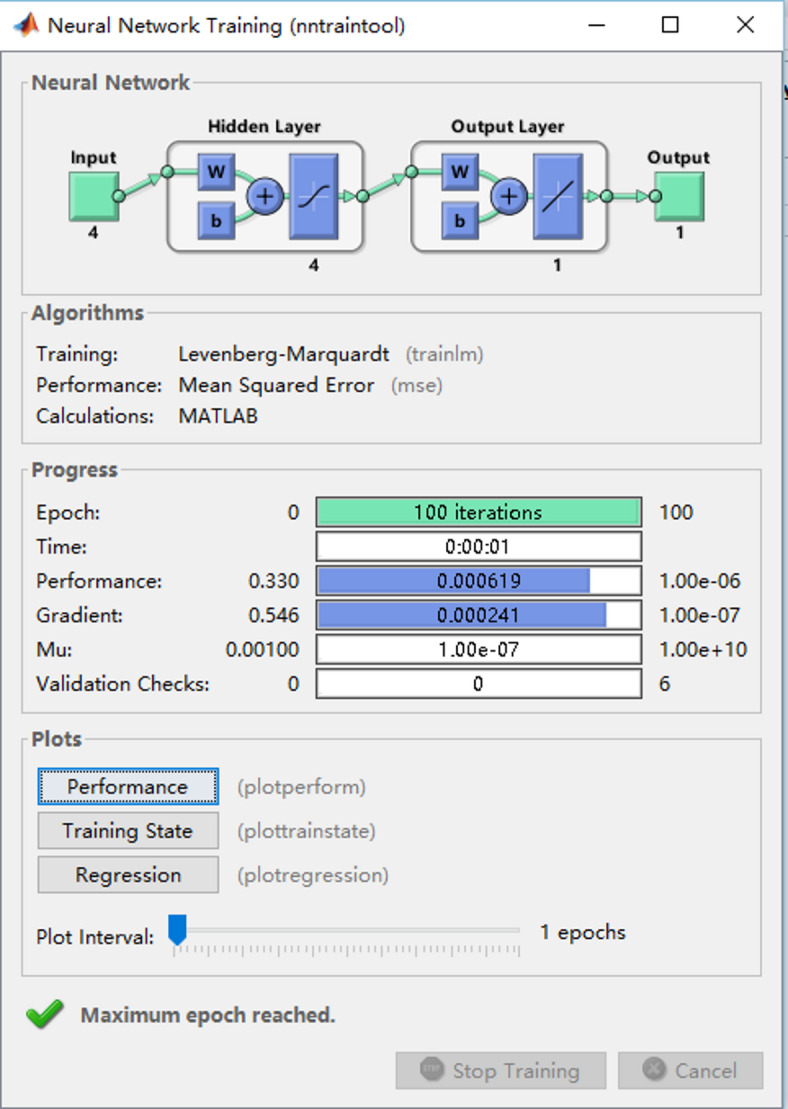
Structure and parameter diagram of the BP neural network.

The influence of each of the above risk factors on the damage caused by hazardous rock tipping collapse can be determined by a neural network. First, the input and output samples of the sample data (see Table 5 for details) are defined: the inclination angles of the hazardous rock joints, the size of hazardous rock, the shape of hazardous rock, and the number of layers of hazardous rock are input into the neural network. The damage caused by hazardous rock tipping collapse is expressed by the critical collapse vibration speed. The critical collapse vibration speed of hazardous rock is output from the BP neural network, which was implemented in MATLAB using the Neural Network Toolbox. A 4-4-1 three-layer neural network is adopted, as shown in Fig. 25, with the tansig() function applied between the input and hidden layers. The purelin() function is used between the hidden and output layers. Finally, the neural network is trained and tested.

Neural network training and testing are carried out using MATLAB software and the Neural Network Toolbox. A total of 19 groups of samples (numbers 2, 3, 4, 5, 8, 10, 11, 12, 13, 14, 15, 16, 18, 19, 21, 25, 26, 27, and 29) in Table 6 are used as training samples, and a total of 10 groups (numbers 6, 9, 7, 17, 24, 20, 1, 28, 22, and 23) in Table 6 are used as test samples. Table 10 shows the results of the network training and test runs.

**Table 10:** Analysis of the projected results.

No	Test sample no	Sectional inclination/ (°)	Hazardous rock dimensions/m	Hazardous rock shape (aspect ratio)	Number of hazardous rock layers	Actual result/(m/s)	Predicted result/(m/s)
6	1	90	1	0.67	10	-0.4141	-0.3784
9	2	90	1	1	5	-0.3954	-0.4124
7	3	90	1	1	15	-0.5227	-0.5388
17	4	90	0.5	1	10	-0.1908	-0.1624
24	5	90	0.5	1	10	-0.2169	-0.1793
20	6	90	0.5	1.5	10	-0.2508	-0.2483
1	7	90	1.5	1	15	-0.7077	-0.7180
28	8	80	0.5	1.5	5	-0.0965	-0.0965
22	9	80	0.5	0.67	10	-0.4961	-0.4963
23	10	80	0.5	1	15	-0.1927	-0.2046

**Fig. 26:**
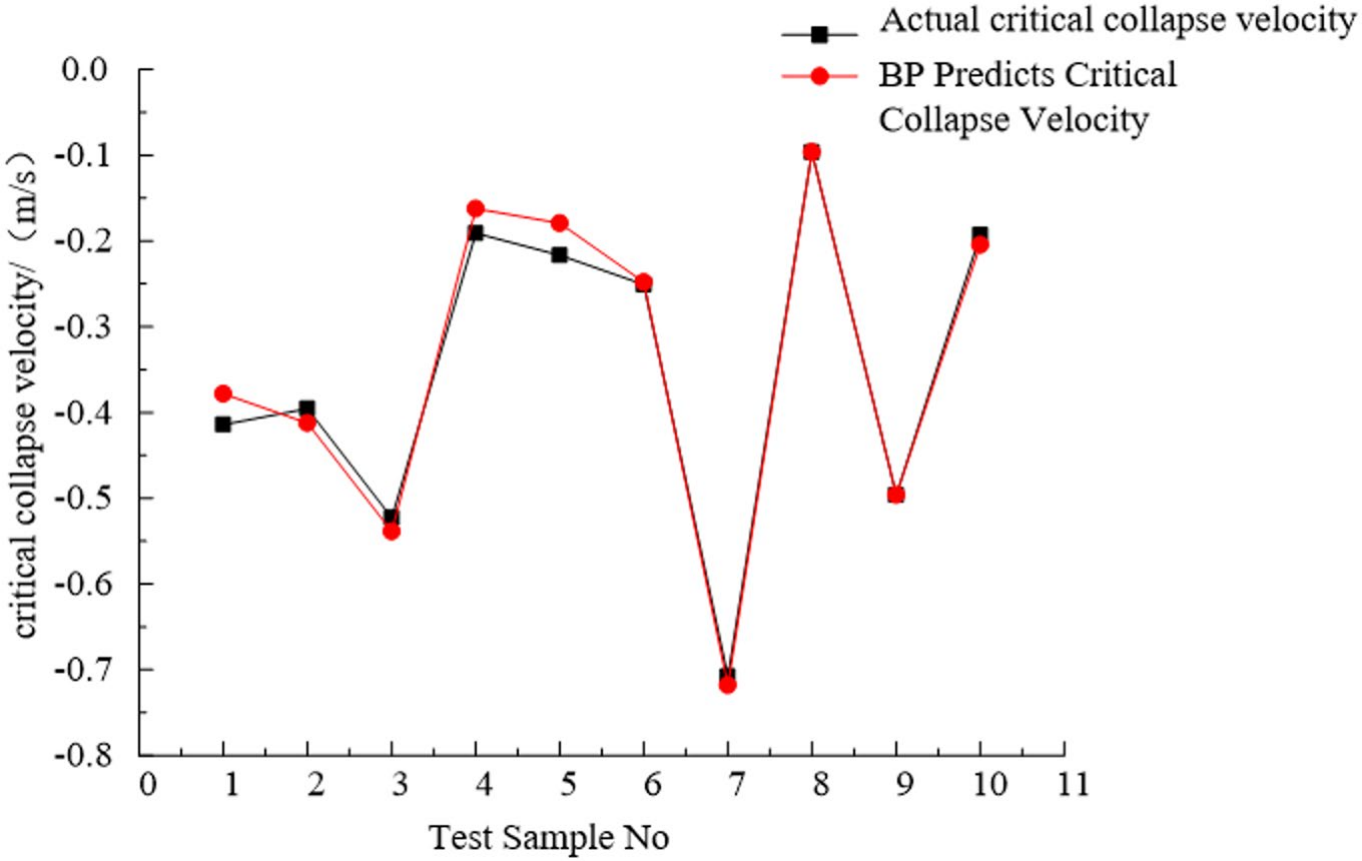
Actual and predicted values of the critical collapse velocity.

The results predicted by the BP network are compared against the actual results by plotting the test sample number on the horizontal axis and the critical collapse vibration velocity on the vertical axis. The results predicted by the BP neural network are shown in Fig. 26.

The relative errors between the BP-predicted and actual critical collapse vibration velocities for the 10 test samples in Fig. 25 are − 8.630%, -4.305%, 3.071%, -14.869%, -17.327%, -0.981%, 1.458%, -0.039%, 0.039%, and 6.152%. The absolute values of these relative errors all fall within the typical range for general engineering errors, and the accuracy is within an acceptable range. Thus, the model established on the basis of the L-M BP neural network to predict the critical collapse vibration speed is reasonable and feasible.

### Model analysis and identification of key risk factors

**Fig. 27:**
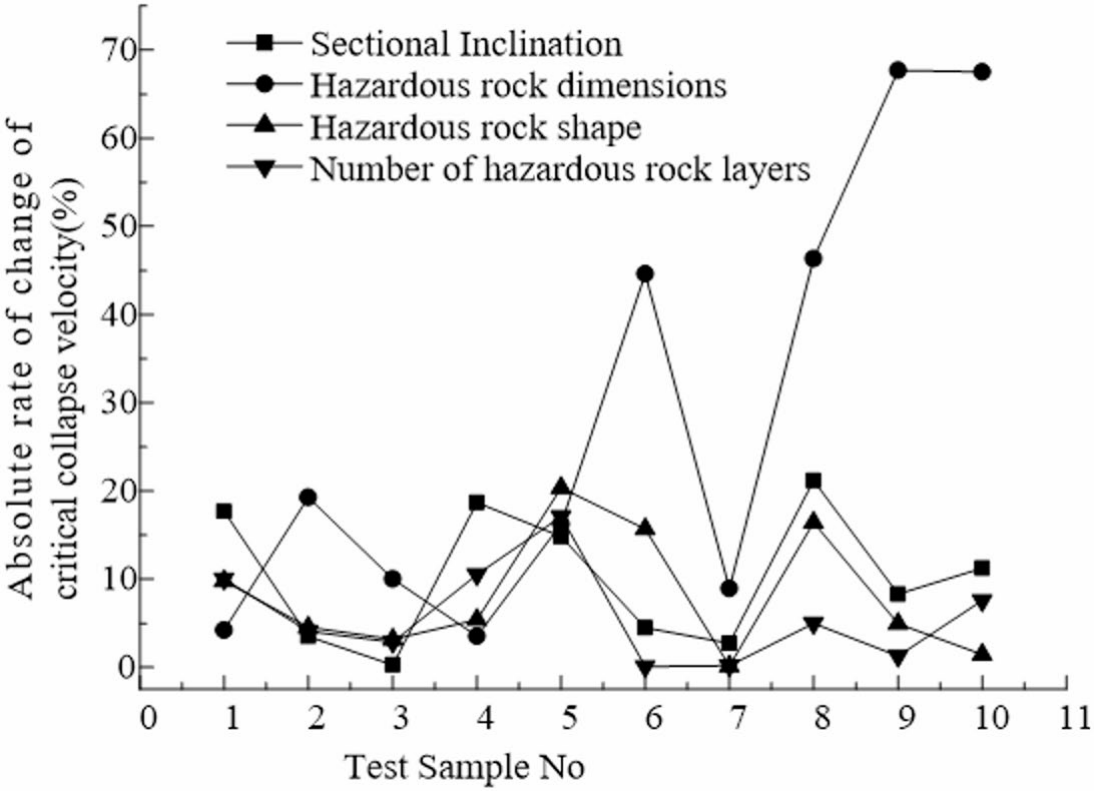
Plot of the absolute change in the critical collapse vibration velocity due to a single factor of 2% increase.

A trained BP neural network model was used to investigate the effects of the four major risk factors mentioned above on collapse damage. The rate of change of the output (critical collapse vibration velocity) was determined by increasing the input of the study factor by 2% while keeping the inputs of the remaining 3 factors constant. The absolute rate of change of the output (critical collapse vibration speed) was calculated by applying the trained BP neural network model to samples 6, 9, 7, 17, 24, 20, 1, 28, 22 and 23. The absolute change in the critical collapse velocity was plotted to facilitate a more intuitive understanding of the importance of each risk factor to the critical collapse velocity, as shown in Fig. 27.

The mean values of the absolute change rates of the joint inclination angle, hazardous rock size, hazardous rock shape and number of hazardous rock layers are sequentially solved to determine their influence on the damage caused by tipping collapse. The results indicate that the mean absolute change rate of the hazardous rock size is 10.281%, the mean absolute change rate of the joint inclination is 28.841%, the mean absolute change rate of the hazardous rock shape is 8.179%, and the mean absolute change rate of the number of hazardous rock layers is 5.866%. Therefore, the order of importance of different risk factors contributing to the collapse of steeply layered hazardous rock cliffs under seismic action is as follows: hazardous rock size > joint inclination > hazardous rock shape > number of hazardous rock layers.

Accordingly, it can be assumed that the largest risk factor for strong earthquake-triggered collapse damage to steep cliff-layered hazardous rocks is the size of the hazardous rock (the degree of fragmentation of the hazardous rock). To reduce the risk of collapse damage, the degree of fragmentation of hazardous rock bodies should be detected and evaluated first in the management of rocky slopes. Serious fragmentation of hazardous rock bodies should be prevented in advance, and corresponding preventive and curative measures should be implemented to avoid threats to the stability of the socioeconomy and the safety of people’s lives and property.

## Discussion

### Comparison with existing stability criteria

The displacement angle threshold of approximately 15° identified in this study provides a practical, geometry-independent metric for collapse warning. This finding aligns with and refines previous research: for instance, Huang et al.^8^ suggested a critical tilt angle of 10°–20° for quasi-static toppling, while Ning et al.^3^ observed that dynamic amplification could reduce the effective stability angle. Our results, derived from dynamic testing, specify a narrower, more conservative threshold under seismic conditions, emphasizing the heightened vulnerability during earthquakes.

The critical collapse vibration velocity ranges (0.10–0.80 m/s, depending on block size) offer a new dynamic criterion. These values are of the same order of magnitude as the peak particle velocities (PPV) associated with rockfall initiation in field studies (e.g., Kuo et al.^28^), but our approach links them directly to identifiable geometric parameters (size, aspect ratio). This advances beyond purely empirical PPV thresholds by incorporating mechanistic, factor-dependent ranges.

### Synthesis of factor interactions and the proposed conceptual framework

The experimental and numerical results reveal a complex, hierarchical control system governing the seismic collapse of steep cliff hazardous rock masses. This interplay can be conceptually synthesized as follows, without reliance on a single visual diagram:

1. Hierarchy of Controlling Factors: The factor importance analysis (Sect.  6.2) established a clear hierarchy: rock size (degree of fragmentation) > joint inclination > rock shape > number of layers. This order indicates that the long-term, preparatory process of weathering (which reduces block size) is the most dominant preconditioning factor, fundamentally weakening the rock mass structure.
2. The ‘Mode-Switch’ Role of Joint Inclination: Joint inclination acts not merely as an influencing parameter but as a decisive ‘kinematic switch’ between the two primary failure modes. For inclinations ≥ 80°, the governing mechanics favor toppling collapse, driven by the overturning moment under seismic excitation. For inclinations ≤ 70°, the mode shifts to sliding collapse, where shear displacement along the steeply inclined planes becomes predominant. This finding corroborates and quantifies the classical kinematic controls discussed in prior literature^11,16^.
3. Nonlinear Modulation by Other Geometric Factors: While the joint inclination sets the potential mode, the actual timing of collapse initiation and the critical vibration velocity are strongly modulated by other factors in a nonlinear manner. For instance: (1) A reduction in rock size (increased fragmentation) drastically accelerates collapse onset, regardless of the joint inclination; (2) Aspect ratio (shape) influences the stability moment arm; values below 1 (flatter blocks) delay toppling, but above a threshold (δ ≈ 1), its effect plateaus; (3) The number of layers exhibits a similar threshold effect, where stability increases up to about 10 layers, beyond which additional layers contribute little.
4. Integration with Dynamic Triggering: The external seismic load provides the dynamic energy required to overcome the prevailing stability margins defined by the above geometric and structural factors. The identified displacement angle (~ 15°) and the size-dependent critical vibration velocity ranges (Table 8) are thus not universal constants, but are the observable outcomes of this factor interplay under seismic forcing.

In summary, the collapse mechanism can be conceptualized as a multi-stage, interactive process: Long-term weathering (reducing rock size) creates a vulnerable mass; the pre-existing joint structure (inclination) dictates the kinematic feasibility of toppling versus sliding; and finally, a seismic event of sufficient intensity, whose critical threshold is further fine-tuned by block shape and layering, triggers the actual collapse. This integrated framework moves beyond single-factor analysis and provides a basis for the multi-parameter predictive modeling demonstrated in this study.

### Limitations and future perspectives

This study, while providing new insights, has several limitations that should be acknowledged. The shaking table tests were necessarily limited in number due to the scale and complexity of the field conditions along the China-Pakistan Highway. Consequently, the predictive model primarily addresses the more frequent toppling collapse mode, with the sliding collapse mode requiring further expansion.

In terms of numerical modeling, the 3DEC approach simulated rock blocks as continuous units with behavior concentrated along pre-defined discontinuities. This simplification efficiently captures structurally controlled failure but does not incorporate the deformation and damage of intact rock between joints, which could offer a more complete picture of stress evolution and fragmentation during collapse.

Methodologically, the scaling laws employed, though based on dynamic similarity principles, may not fully replicate all scale-dependent behaviors of in-situ rock masses. The boundary conditions (rigid base, fixed sides) and the use of sinusoidal seismic inputs, while representative, simplify the energy dissipation and spectral complexity of real slopes and earthquakes.

Future work should include more diverse laboratory experiments and extend the analysis to sliding collapse mechanisms. Expanding the research to other segments of the China-Pakistan Highway and similar corridors (e.g., China-Kyrgyzstan-Uzbekistan Highway) would enhance generalizability. International collaboration with Central Asian scholars could yield region-specific management strategies. Furthermore, comparative studies with other machine learning models (e.g., SVM, Random Forest) would help validate and potentially improve the predictive framework established here. Finally, incorporating more realistic boundary conditions, scaled natural ground motions, and constitutive models that account for intact rock damage would advance the numerical simulation towards a more comprehensive collapse analysis.

## Conclusions

This study systematically investigates failure modes, critical collapse thresholds, and risk factors through an integrated approach combining scaled shaking table tests and 3DEC discrete element modeling, focusing on hazardous rock formations along the China-Pakistan Highway. Combined with a field investigation, the concept of the displacement angle is introduced to conduct an in-depth study on the collapse behaviour of hazardous rock groups, focusing on four factors: the degree of blockiness, joint inclination, height, and earthquake intensity. A 3-D discrete-element numerical simulation method is combined with BP artificial neural network theory to predict the instability mode of steep cliff hazardous rock under strong earthquakes, identify the tipping critical collapse state and risk factors for tipping collapse, promote further theoretical research in strong earthquake-induced steep cliff hazardous rock collapse disasters and predict and control the risks of hazardous rock collapse disasters. The main research results and conclusions of the paper are as follows:

1. As the height of the block group, joint inclination and vibration intensity gradually increase, the collapse angle of hazardous rock groups does not monotonically increase or decrease. Instead, it exhibits fluctuations, alternating between decreasing and then increasing or increasing and then decreasing. In addition, the value of the collapse displacement angle of the hazardous rock body when the side length changes shows that the collapse angle increases with the side length of the hazardous rock group, with a clear positive correlation. The findings suggest that the collapse angle threshold of hazardous rock is approximately 15°, the closest connection between the degree of rock fragmentation and the degree of weathering of the hazardous rock body has the most decisive role in the collapse damage of hazardous rock bodies.
2. The effects of different hazardous rock joint inclinations, sizes, shapes, and numbers of layers on the destabilization and tilting collapse behaviours of laminated hazardous rocks in steep cliffs were investigated. The deformation destabilization modes and the law of critical collapse were summarised. The destabilization modes of hazardous rocks include tipping collapse and slipping collapse; the dip angle of joints is a controlling factor for the destabilization mode of laminated hazardous rocks.
3. By using BP neural network nonlinear mapping and adaptive learning to train and test numerical test samples, a neural network model capable of predicting the deformation and instability patterns of steep cliff-layered hazardous rocks was established, and the risk of dumping-type collapse of hazardous rocks was assessed.
4. The critical collapse velocity for tipping-type failures was introduced to estimate the onset of collapse under strong earthquakes, and the effects of four major factors were analysed: the onset moment of the collapse damage advanced with increasing joint inclination angle; the size of the hazardous rock and the time of the collapse were positively correlated, and the more fragmented the hazardous rock was, the more rapidly the collapse occurred; the onset moment of the collapse damage of the hazardous rock body decreased and then remained unchanged as the hazardous rock shape increased; and the onset moment of the collapse damage of the hazardous rock body decreased and then remained unchanged as the number of hazardous rock layers increased. The moment of collapse damage of the hazardous rock body tended to increase and then remained unchanged with increasing number of hazardous rock layers.
5. The change rules of the critical collapse vibration velocity for different hazardous rock joint inclination angles, hazardous rock sizes, hazardous rock shapes and numbers of hazardous rock layers on dumped collapse hazardous rock were determined, the main factors affecting the critical collapse vibration velocity of hazardous rock were determined, and the threshold value of the critical collapse vibration velocity of hazardous rock under different hazardous rock sizes was determined.

The displacement angle method proposed in this paper, the 3DEC numerical simulation classification of hazardous rock mass collapse patterns, the BP neural network model for predicting hazardous rock mass instability patterns, and the risk factor identification model provide a sound predictive theoretical foundation and valuable modeling references for hazardous rock mass management, particularly for the management of hazardous rock mass collapses along the China-Pakistan Highway (within China). Future studies will build upon these findings to address the aforementioned limitations and expand the predictive framework to a broader range of collapse scenarios and geographic regions.

## Data Availability

The datasets generated and analysed during the current study, including raw shaking table test results, processed DIC data, 3DEC simulation input files, and MATLAB scripts for the BP neural network model, are not publicly available due to ongoing research but are available from the corresponding author (Naman Maimaiti, namanjan@163.com) on reasonable request. Example datasets and code snippets are provided in the Supplementary Information accompanying this manuscript.
